# UAV Aeromagnetic Path Planning in Complex Terrain Based on a Q-Learning-Assisted Multi-Strategy Starfish Optimization Algorithm

**DOI:** 10.3390/biomimetics11050326

**Published:** 2026-05-07

**Authors:** Sihan Yuan, Zhipeng Li, Junjie Zhang

**Affiliations:** School of Mechanical and Electrical Engineering, Chengdu University of Technology, Chengdu 610059, China; 202319070324@stu.cdut.edu.cn (S.Y.); 202319150125@stu.cdut.edu.cn (J.Z.)

**Keywords:** aeromagnetic survey, metaheuristic algorithm, path planning, Q-learning, UAV

## Abstract

Low-altitude terrain-following flight is essential for obtaining high-quality data in unmanned aerial vehicle (UAV) aeromagnetic surveys, but achieving efficient and safe path planning within complex terrains remains challenging. To address this issue, a Q-learning-assisted multi-strategy Starfish Optimization Algorithm (QMSFOA) is proposed for offline path planning. The proposed algorithm integrates four improvement strategies: (1) employing a Sobol sequence combined with Refraction Opposition-based Learning for population initialization to enhance population diversity; (2) adopting a hybrid adaptive differential mutation mechanism to improve search efficiency; (3) utilizing Q-learning to intelligently schedule optimization modes, thereby accelerating convergence speed; (4) introducing an adaptive t-distribution elite perturbation strategy to refine convergence accuracy. Experimental results on the CEC-2022 benchmark suite indicate that QMSFOA achieves the best convergence accuracy on nine functions and exhibits a superior performance across most metrics compared with the competing algorithms. Simulation experiments of aeromagnetic surveys in complex 3D terrains demonstrate that paths planned by QMSFOA satisfy kinematic and obstacle avoidance constraints while reducing path costs by approximately 25% compared with the standard Starfish Optimization Algorithm (SFOA). Additionally, the standard deviation is reduced by one to two orders of magnitude compared with the competing algorithms. These results demonstrate that the proposed method provides an efficient, reliable, and intelligent solution for high-precision UAV geophysical exploration in complex environments.

## 1. Introduction

Aeromagnetic surveying, an efficient geophysical exploration technique, reveals subsurface geological structures by measuring variations in the Earth’s magnetic field [1,2,3]. It is extensively applied in mineral resource exploration, geological mapping, and deep structural interpretation, as well as in groundwater assessment, geothermal energy evaluation, and environmental pollution monitoring [4,5,6,7,8]. Traditional aeromagnetic surveys primarily rely on manned aircraft, which face operational constraints in maintaining low-altitude flight over complex mountainous terrain, resulting in elevated survey altitudes and degraded data resolution [9]. In recent years, with the rapid advancement of UAV technology, its application in geophysical exploration has become increasingly widespread. While fixed-wing UAVs possess significant advantages for large-scale surveys in flat regions, they face challenges related to kinematic constraints when operating in complex mountainous terrain [10]. In contrast, rotary-wing UAVs, with their vertical take-off and landing (VTOL) and hovering capabilities, have emerged as an ideal platform for executing high-precision magnetic survey tasks in complex environments [11].

According to potential field theory, the amplitude of magnetic anomalies decays as the inverse cube of the distance between the observation point and the magnetic source [12]. To capture weak magnetic signals associated with deep-seated mineralization, UAVs are required to perform high-precision low-altitude terrain-following flight [13,14]. Feng et al. [15] indicated that efficient terrain-following capability is a fundamental prerequisite for achieving nap-of-the-earth detection. In rugged terrain with severe topographic relief, UAVs must also exhibit strong obstacle avoidance capabilities to effectively maneuver around abrupt peaks and obstacles while maintaining terrain following. Furthermore, aeromagnetic surveys have stringent requirements for flight attitude stability. Schmidt et al. [16] reported that flight state stability is directly correlated with the fidelity of aeromagnetic data. To suppress noise fluctuations induced by abrupt attitude variations, UAVs must maintain a constant speed and smooth trajectories, avoiding frequent pitch adjustments caused by overly aggressive terrain-following demands. This viewpoint was further supported by Kaub et al. [17], who emphasized in their accuracy assessment that optimizing flight strategies is essential to minimize errors from sensor attitude variations. However, flight strategies relying on real-time obstacle avoidance often require frequent maneuvers, thereby introducing significant magnetic interference. To mitigate such interference, aeromagnetic missions typically utilize offline global path planning to provide a smooth, stable, and safe reference trajectory for low-altitude terrain-following operations [18].

To address path planning problems under complex constraints, early studies predominantly employed traditional algorithms such as A* [19], Artificial Potential Field (APF) [20], and Rapidly exploring Random Tree (RRT) [21]. Although these methods have mature theoretical foundations, they exhibit limitations in high-dimensional and complex nonlinear terrains. Graph-search approaches such as A* suffer from state-space explosion as the resolution of environmental discretization increases, particularly in 3D environments. APF is prone to local minima in dense obstacle distributions, leading to planning failure. Sampling-based planners such as RRT show reduced convergence efficiency in high-dimensional spaces. In response to these limitations, metaheuristic algorithms [22] with strong global search capabilities have become an important research focus. A bibliometric analysis by Wu et al. of UAV path-planning research over the past two decades indicated that swarm intelligence optimization algorithms have gradually surpassed traditional methods [23]. Due to their robustness in multimodal obstacle-avoidance problems, these algorithms are now widely adopted for trajectory planning in complex environments. In this progression, various optimization techniques have provided numerous path-planning solutions. Early representative algorithms include Particle Swarm Optimization (PSO) [24], Ant Colony Optimization (ACO) [25], and Artificial Bee Colony (ABC) [26]. Subsequently, other widely recognized algorithms emerged, such as Grey Wolf Optimizer (GWO) [27], Whale Optimization Algorithm (WOA) [28], and Harris Hawks Optimization (HHO) [29]. More recently, to cope with higher-dimensional nonlinear constraints, novel bio-inspired algorithms have been introduced, including Sparrow Search Algorithm (SSA) [30], Dung Beetle Optimizer (DBO) [31], Pelican Optimization Algorithm (POA) [32], and Black-winged Kite Algorithm (BKA) [33].

According to the No Free Lunch (NFL) theorem, no single optimization algorithm can guarantee an optimal performance across all problems [34]. This implies that complex optimization problems, characterized by specific solution space features, demand targeted improvements and adaptations. Following this rationale, researchers have conducted extensive investigations into algorithmic improvements. Yang et al. [35] proposed a multi-strategy Sparrow Search Algorithm (MSSA), which adopts strategies such as Levy flight and Cauchy mutation to effectively enhance the algorithm’s ability to escape local optima. Qiu et al. [36] introduced a multi-strategy improved Pelican Optimization Algorithm (IPOA), incorporating iterative chaotic mapping and refraction opposition-based learning to significantly boost global search capability. Tu et al. [37] developed an enhanced Dung Beetle Optimizer (EDBO), utilizing strategies such as Sobol sequence initialization and adaptive t-distribution mutation to markedly improve obstacle avoidance performance in complex environments. Despite the demonstrated effectiveness of these improved algorithms in standard path planning tasks, they primarily optimize for path length and safety, without fully adapting to the unique physical constraints of aeromagnetic surveys. If they are directly applied to aeromagnetic survey missions, the generated trajectories often fail to satisfy the specialized requirements.

In the search for a more promising solver to address these challenges, the Starfish Optimization Algorithm (SFOA) proposed by Zhong et al. [38] has demonstrated promising application prospects. In comparative benchmarks against 100 mainstream optimizers, this algorithm exhibited a superior global optimization performance, indicating its strong potential for handling high-dimensional, complex problems. However, when applied to the challenges of complex terrain constraints and large-scale search spaces in aeromagnetic surveys, the standard SFOA still reveals inherent limitations such as loss of population diversity, slow convergence speed, and susceptibility to local optima.

To overcome these limitations and meet stringent path quality requirements in aeromagnetic surveys, this study proposes a Q-learning-assisted multi-strategy Starfish Optimization Algorithm (QMSFOA). The main contributions of this paper are as follows:

(1) Q-learning-assisted Multi-strategy Starfish Optimization Algorithm (QMSFOA): To address the limitations of the standard SFOA, QMSFOA integrates four improvement strategies into its algorithmic framework. This integration significantly enhances the algorithm’s global optimization accuracy and robustness when solving high-dimensional complex problems.
(2) Application to Aeromagnetic Path Planning in Complex Terrain: QMSFOA is applied to high-fidelity simulation scenarios of aeromagnetic survey operations. Through rigorous benchmarking against six other metaheuristic algorithms, including the standard SFOA, the superiority and robustness of QMSFOA are verified.

The remainder of this paper is organized as follows: Section 2 briefly introduces the fundamental principles of SFOA. Section 3 details the specific implementation of the improvement strategies in QMSFOA. Section 4 formulates a mathematical model for 3D UAV aeromagnetic path planning. Section 5 presents a multi-dimensional performance analysis of QMSFOA using the CEC2022 benchmark suite and the Wilcoxon rank-sum test. Section 6 applies QMSFOA to simulation experiments for aeromagnetic survey path planning in complex mountainous environments to assess its practical value. Finally, Section 7 concludes the paper and discusses future research directions.

## 2. Starfish Optimization Algorithm (SFOA)

SFOA [38] is inspired by the exploration, predation, and regeneration behaviors of starfish. During the exploration phase, the algorithm adopts a hybrid search mechanism that combines five-dimensional (5D) and one-dimensional (1D) patterns to enhance search efficiency. In the exploitation phase, it utilizes a parallel two-directional search approach driven by the information of two individuals to facilitate robust global convergence. The core mathematical modeling of SFOA is detailed as follows.

### 2.1. Population Initialization

During the initialization phase, a population of N starfish individuals is randomly generated within the constraints of the search space. The position of the i-th individual in the j-th dimension, denoted as $X_{ij}$, is defined as follows:

(1) $$X_{ij}=l_{j}+r\cdot \left(u_{j}-l_{j}\right),\;i=1,2,\ldots ,N,\;j=1,2,\ldots ,D$$

where $r\in \left(0,1\right)$ is a random number; $u_{j}$ and $l_{j}$ represent the upper and lower bounds of the j-th dimension, respectively; and D denotes the dimensionality of the optimization problem.

### 2.2. Exploration Phase

The exploration phase of SFOA mimics the behavior of starfish, which utilize the photosensitive eyes at the tips of their arms to perceive the surrounding environment. Based on the dimensionality D of the optimization problem, the algorithm employs either a 5D or a 1D search mode. This design is derived from the five-arm structure of a starfish.

When the dimension of the optimization problem exceeds 5 $\left(D>5\right)$, the 5D search mode is adopted to emulate the starfish using five arms to search simultaneously. In this mode, the algorithm randomly selects five dimensions for updating during each iteration. The mathematical model is expressed as:

(2) $$\left\{\begin{array}{ll} Y_{i,p}^{T}=X_{i,p}^{T}+a_{1}\left(X_{best,p}^{T}-X_{i,p}^{T}\right)cos\theta , & r\leq 0.5\\ Y_{i,p}^{T}=X_{i,p}^{T}-a_{1}\left(X_{best,p}^{T}-X_{i,p}^{T}\right)sin\theta , & r>0.5 \end{array}\right.$$

where $Y_{i,p}^{T}$ and $X_{i,p}^{T}$ represent the updated candidate position and the current position, respectively, and $X_{best,p}^{T}$ denotes the position of the current global best solution in the p-th dimension. The parameters $a_{1}$ and θ are calculated as follows:

(3) $$a_{1}=\left(2r-1\right)$$

(4) $$\theta =\frac{\pi }{2}\cdot \frac{T}{T_{max}}$$

where T is the current iteration number, and $T_{max}$ represents the maximum number of iterations.

When the dimension of the optimization problem does not exceed 5 $\left(D\leq 5\right)$, the 1D search mode is employed. Here, each starfish uses only one arm for searching while leveraging the position information of other individuals. In this case, the algorithm randomly selects a single dimension p for updating, formulated as:

(5) $$Y_{i,p}^{T}=E_{t}X_{i,p}^{T}+A_{1}\left(X_{k_{1},p}^{T}-X_{i,p}^{T}\right)+A_{2}\left(X_{k_{2},p}^{T}-X_{i,p}^{T}\right)$$

where $X_{k_{1},p}^{T}$ and $X_{k_{2},p}^{T}$ correspond to the p-th dimension positions of two distinct individuals randomly selected from the population; $A_{1},A_{2}\in \left(-1,1\right)$ are random numbers; and $E_{t}$ represents the starfish’s energy, calculated as:

(6) $$E_{t}=\frac{T_{max}-T}{T_{max}}\cos\theta$$

In both search modes, if the updated candidate position exceeds the search space boundaries, the original position is retained. This boundary control mechanism is mathematically expressed as:

(7) $$X_{i,p}^{T+1}=\left\{\begin{array}{ll} Y_{i,p}^{T}, & l_{b,p}\leq Y_{i,p}^{T}\leq u_{b,p}\\ X_{i,p}^{T}, & otherwise \end{array}\right.$$

where $l_{b,p}$ and $u_{b,p}$ represent the lower and upper bounds of the p-th dimension, respectively.

### 2.3. Exploitation Phase

The exploitation phase simulates the predation and regeneration behaviors of starfish to identify the global optimum.

A parallel two-directional search approach is incorporated to mimic the predation process. This approach utilizes the information of the current global best individual and other randomly selected individuals to guide the search. First, the distance vectors $d_{m}$ between the current global best solution $X_{best}^{T}$ and five randomly selected individuals $X_{m_{p}}^{T}$ are computed as:

(8) $$d_{m}=\left(X_{best}^{T}-X_{m_{p}}^{T}\right),\;m=1,2,\ldots ,5$$

Subsequently, two component vectors, $d_{m1}$ and $d_{m2}$, are randomly selected from these five distance vectors to update the individual’s position:

(9) $$Y_{i}^{T}=X_{i}^{T}+r_{1}d_{m1}+r_{2}d_{m2}$$

where $r_{1,}r_{2}\in \left(0,1\right)$ are random numbers. This parallel two-directional search approach not only propels individuals toward better positions but also effectively maintains population diversity within local regions.

To prevent search stagnation, SFOA introduces a regeneration mechanism specifically for the last individual $\left(i=N\right)$ in the population. The mathematical model is defined as:

(10) $$Y_{i}^{T}=\exp\left(-\frac{T\times N}{T_{max}}\right)X_{i}^{T}$$

**Figure 1:**
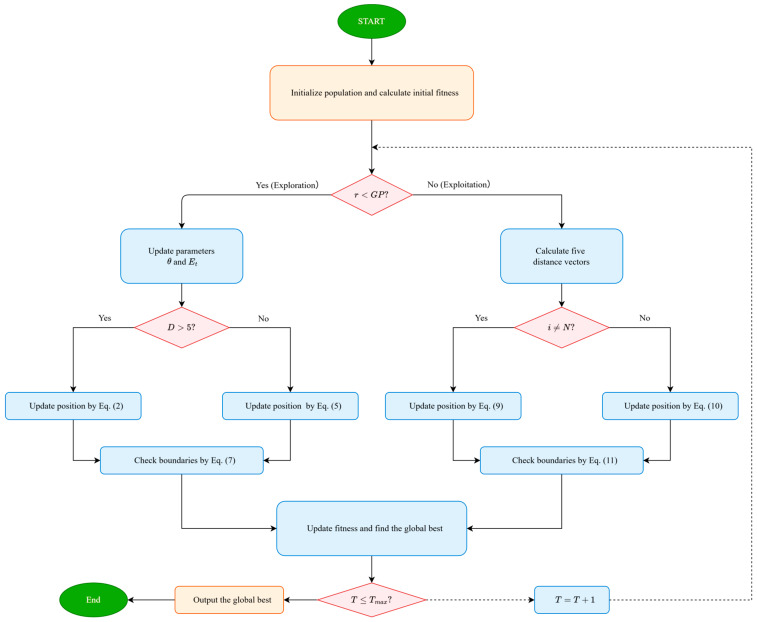
The flowchart of SFOA.

If a candidate position generated during the exploitation phase exceeds the variable boundaries, it is constrained to the boundary values. This process is mathematically expressed as:

(11) $$X_{i}^{T+1}=\left\{\begin{array}{cc} Y_{i}^{T}, & l_{b}\leq Y_{i}^{T}\leq u_{b}\\ l_{b}, & Y_{i}^{T}<l_{b}\\ u_{b}, & Y_{i}^{T}>u_{b}\;\;\;\; \end{array}\right.$$

where $l_{b}$ and $u_{b}$ represent the lower and upper bounds of the variable, respectively. The flowchart of SFOA is presented in Figure 1.

## 3. Q-Learning-Assisted Multi-Strategy Starfish Optimization Algorithm (QMSFOA)

### 3.1. Population Initialization via Sobol Sequence and Refraction Opposition-Based Learning

In swarm intelligence optimization algorithms, the ergodicity and diversity of the initial population directly determine search efficiency and convergence accuracy. The standard SFOA employs pseudo-random number generators for initialization. This purely randomized approach often leads to an uneven distribution in the solution space, resulting in individual clustering and the potential neglect of promising regions. To overcome this limitation, a hybrid initialization strategy combining the Sobol sequence [39] and Refraction Opposition-Based Learning (ROBL) [40] is proposed.

#### 3.1.1. Sobol Sequence Strategy

The Sobol sequence is a deterministic low-discrepancy sequence. Compared to pseudo-random initialization methods, the Sobol sequence exhibits superior uniformity in multi-dimensional hypercubes. Leveraging this sequence for initialization significantly improves the uniformity of the population distribution, laying a robust foundation for global search.

Assuming a population size of N and problem dimensionality D, the initial position $X_{ij}$ of the i-th individual in the j-th dimension generated by the Sobol sequence is calculated as:

(12) $$X_{ij}=l_{j}+s_{i,j}\cdot \left(u_{j}-l_{j}\right),\;i=1,\ldots ,N,\;j=1,\ldots ,D$$

where $l_{j}$ and $u_{j}$ represent the lower and upper bounds of the j-th dimension, respectively, and $s_{ij}\in \left[0,1\right]$ is the quasi-random number generated by the Sobol sequence. Through this mapping, the algorithm establishes a uniformly distributed initial solution set within the search space.

**Figure 2:**
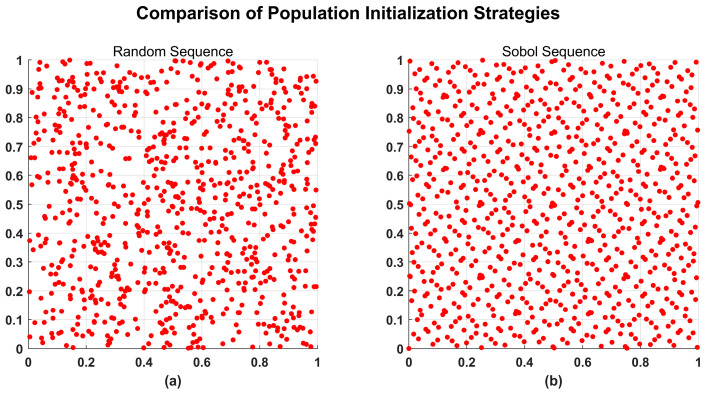
Comparison of population initialization strategies. (**a**) Pseudo-random sequence. (**b**) Sobol sequence.

To demonstrate the superiority of the Sobol sequence, Figure 2 compares the distribution of 800 sample points generated by a pseudo-random sequence and a Sobol sequence in a 2D space. As observed, the pseudo-randomly generated point cloud (Figure 2a) exhibits distinct clustering and search blind spots. In contrast, the point cloud generated by the Sobol sequence (Figure 2b) presents a highly uniform distribution, effectively covering the entire solution space. This uniformity significantly enhances the probability of capturing promising regions during the early exploration phase.

#### 3.1.2. Refraction Opposition-Based Learning (ROBL)

**Figure 3:**
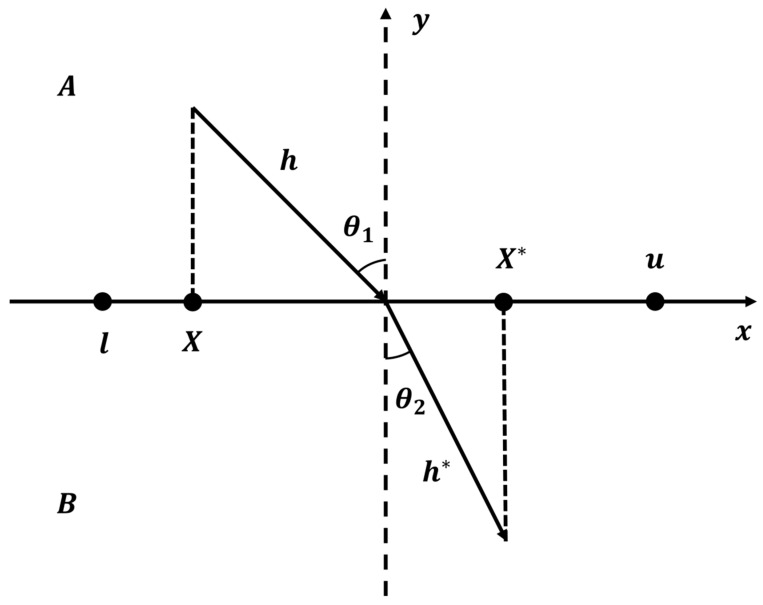
Refraction Opposition-Based Learning.

Although the Sobol sequence ensures spatial uniformity, it cannot guarantee the quality of initial solutions. Therefore, Refraction Opposition-Based Learning (ROBL) [40] is further incorporated to enhance population diversity and improve the quality of candidate solutions. In contrast to traditional Opposition-Based Learning (OBL) [41], which generates candidate solutions strictly at center-symmetric positions, ROBL integrates the physical principle of light refraction. By introducing a scaling factor to adjust the position of the opposite solution, ROBL exhibits stronger global exploration capabilities. The principle is illustrated in Figure 3.

As illustrated in Figure 3, consider a search space bounded by $\left[l,u\right]$, and the origin O is located at the midpoint of the interval. Let X be the current candidate solution and $X^{*}$ be its refraction-based opposite solution. According to Snell’s law, when a light ray travels from medium A into medium B, its path satisfies the following geometric relationship:

(13) $$\gamma =\frac{\sin\theta_{1}}{\sin\theta_{2}}=\frac{\left(\left(l+u\right)/2-X\right)/h}{\left(X^{*}-\left(l+u\right)/2\right)/h^{*}}$$

where γ is the refractive index; $\theta_{1}$ and $\theta_{2}$ denote the angle of incidence and the angle of refraction, respectively; and h and $h^{*}$ represent the lengths of the incident and refracted rays.

Let $\eta =h/h^{*}$ denote the scaling factor. By substituting this into Equation (12), the mathematical expression for the opposite solution $X^{*}$ can be derived as:

(14) $$X^{*}=\frac{l+u}{2}+\frac{l+u}{2\eta \gamma }-\frac{X}{\eta \gamma }$$

Generalizing this mechanism to the D-dimensional population initialization, for an initial individual $X_{ij}$ generated by the Sobol sequence, its refraction opposite solution $X_{ij}^{*}$ is calculated as:

(15) $$X_{i,j}^{*}=\frac{l_{j}+u_{j}}{2}+\frac{l_{j}+u_{j}}{2\eta \gamma }-\frac{X_{i,j}}{\eta \gamma },\;i=1,\ldots ,N;\;j=1,\ldots ,D$$

Finally, the algorithm adopts a greedy selection strategy: the N individuals generated by the Sobol sequence and the N individuals generated by ROBL are combined and sorted based on fitness values. The top N individuals with the best fitness are then retained as the final initial population.

### 3.2. Hybrid Adaptive Differential Mutation Mechanism

When applied to complex, high-dimensional, and multi-modal environments, the standard SFOA often suffers from two critical limitations: high-dimensional search inefficiency and a lack of directional guidance. Differential Evolution (DE) [42], with its unique differential mutation mechanism, leverages distance information between individuals to guide the search direction. It has been proven to be an effective strategy for enhancing population diversity and escaping local optima. However, traditional DE, with its fixed control parameters and single mutation strategy, often struggles to maintain the balance between global exploration and local exploitation. 

To address these limitations, QMSFOA incorporates a hybrid adaptive differential mutation mechanism, integrating the strategy pool adaptation philosophy of Self-adaptive Differential Evolution (SaDE) [43] and the parameter adaptive control concept of Adaptive Differential Evolution with Optional External Archive (JADE) [44]. This mechanism comprises two core components:

(1) During the global exploration phase, an adaptive dimension selection strategy is introduced, where the proportion of mutated dimensions is dynamically adjusted based on historical success records.
(2) During the regeneration phase, a hybrid strategy reconstruction mechanism is established. By using a probability-guided strategy pool and adaptive Cauchy step sizes, stagnant individuals are intelligently reconstructed to escape from local optima.

#### 3.2.1. Adaptive Dimension Selection Strategy

In the global exploration mode of the standard SFOA, the fixed-dimension update strategy limits the exploration efficiency within high-dimensional solution spaces. To address this limitation, an adaptive parameter $\mu_{CR}$ is introduced to dynamically control the proportion of dimensions in position updates.

For the i-th individual at generation t, the dimension selection rate $CR_{i,t}$ is generated from a normal distribution with mean $\mu_{CR,t}$ and standard deviation $\sigma_{CR}$:

(16) $$CR_{i,t}∼N\left(\mu_{CR,t},\sigma_{CR}^{2}\right)$$

To ensure validity, if $CR_{i,t}$ falls outside the interval (0,1], it is truncated to the nearest boundary. Based on $CR_{i,t}$, the number of dimensions $p_{i,t}$ participating in the mutation for the current individual is determined as:

(17) $$p_{i,t}=\max\left(1,\;round\left(CR_{i,t}\cdot D\right)\right)$$

where D represents the total dimensionality of the problem. Subsequently, the algorithm randomly selects $p_{i,t}$ dimensions from the search space to update the individual’s position.

To achieve adaptive parameter adjustment, the learning period LP is defined. $\mu_{CR}$ is updated based on historical success records within the current learning period. The update rule for the mean value in the next generation is:

(18) $$\mu_{CR,t+1}=\left(1-c\right)\cdot \mu_{CR,t}+c\cdot {mean}_{A}\left(S_{CR}\right)$$

where c is the learning rate; $S_{CR}$ denotes the set of CR values that successfully generated better fitness in the current period; and ${mean}_{A}\left(\cdot \right)$ denotes the arithmetic mean.

#### 3.2.2. Hybrid Strategy Reconstruction Mechanism

To assist stagnant individuals in escaping the basins of attraction of local optima, QMSFOA reconstructs the regeneration phase by increasing the number of regenerated individuals from 1 to 2 and introducing a mechanism that combines Cauchy-based step size adaptation with probability-guided strategy selection.

**Figure 4:**
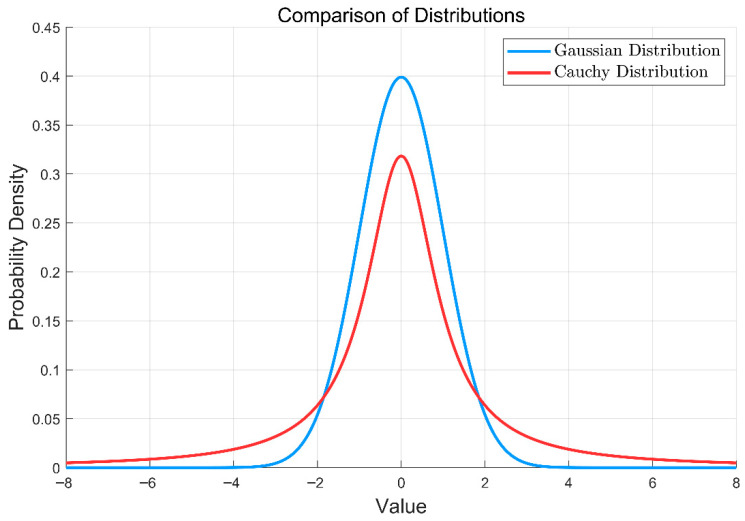
The probability densities of the Cauchy and Gaussian distributions.

The probability density function of the Cauchy distribution is defined as:

(19) $$f\left(x;x_{0},\gamma \right)=\frac{1}{\pi \gamma \left[1+{\left(\frac{x-x_{0}}{\gamma }\right)}^{2}\right]},\;x\in \left(-\infty ,+\infty \right)$$

where $x_{0}$ is the location parameter and γ is the scale parameter. Figure 4 presents a comparison between the probability densities of the Cauchy and Gaussian distributions.

As shown in Figure 4, compared to the Gaussian distribution, the Cauchy distribution exhibits significant heavy-tail characteristics. This implies a higher probability of generating large mutation step sizes far from the center, endowing the algorithm with a stronger capability to escape local optima.

Therefore, the Cauchy distribution is employed to generate the scaling factor $F_{i,t}$ for the i-th individual at generation t:

(20) $$F_{i,t}∼C\left(\mu_{F,t},\gamma_{F}\right)$$

where $\mu_{F,t}$ corresponds to the location parameter and $\gamma_{F}$ to the scale parameter. If $F_{i,t}$ falls outside the interval (0,2], boundary constraints are applied. To encourage the generation of effective large-step mutations, the Lehmer mean is utilized to update $\mu_{F,t}$:

(21) $$\mu_{F,t+1}=\left(1-c\right)\cdot \mu_{F,t}+c\cdot {mean}_{L}\left(S_{F}\right)$$

where $S_{F}$ denotes the set of F values that successfully generated better fitness in the current period, and ${mean}_{L}\left(\cdot \right)$ represents the Lehmer mean, calculated as:

(22) $${mean}_{L}\left(S_{F}\right)=\frac{\sum_{F\in S_{F}}F^{2}}{\sum_{F\in S_{F}}F}$$

To balance population diversity and convergence speed, a strategy pool comprising two canonical mutation strategies, namely “DE/rand/1” and “DE/current-to-best/1”, is constructed:

(1) Strategy 1: DE/rand/1:
  (23) $$V_{i,t}=X_{r1,t}+F_{i,t}\cdot \left(X_{r2,t}-X_{r3,t}\right)$$
(2) Strategy 2: DE/current-to-best/1:

(24) $$V_{i,t}=X_{i,t}+F_{i,t}\cdot \left(X_{best,t}-X_{i,t}\right)+F_{i,t}\cdot \left(X_{r1,t}-X_{r2,t}\right)$$

where $X_{r1,t}$, $X_{r2,t}$, $X_{r3,t}$ are distinct individuals randomly selected from the non-regenerated population, and $X_{best,t}$ denotes the best individual in the current generation.

Let $P_{i,t}$ denote the probability that the i-th regenerated individual selects Strategy 1 at generation t and the probability of selecting Strategy 2 is $1-P_{i,t}$. For each individual undergoing regeneration, one of these strategies is selected based on probability to generate the trial vector $V_{i,t}$ for position updating.

The selection probability $P_{i,t}$ is adaptively adjusted based on the success rate $R_{k}$ observed within the learning period LP. Let $ns_{k}$ and $nf_{k}$ represent the number of successes and failures, respectively, for strategy $k\left(k=1,2\right)$ during the current period. The success rate $R_{k}$ is defined as:

(25) $$R_{k}=\frac{ns_{k}}{ns_{k}+nf_{k}}$$

The update rule for the probability $P_{i,t+1}$ in the next generation is expressed as:

(26) $$P_{i,t+1}=\frac{R_{1}}{R_{1}+R_{2}}$$

Through this mechanism, QMSFOA adaptively adjusts the mutation dimension ratio, step size, and regeneration strategy. This enables efficient optimization in complex high-dimensional environments.

### 3.3. Q-Learning-Based Intelligent Mode Scheduling Strategy

In standard SFOA, the probability of executing exploration or exploitation is fixed. This static switching mechanism neglects the varying demands of the population across different evolutionary stages, leading to inefficient allocation of computational resources. To address this issue, Q-learning [45] is introduced to replace this selection process.

Q-learning is a model-free reinforcement learning method where an agent learns an optimal policy by iteratively updating a Q-table based on environment interactions. In this study, the iterative optimization process of QMSFOA is formulated within a learning framework based on the Markov Decision Process (MDP), comprising three core elements: state space, action space, and reward mechanism.

#### 3.3.1. State Space

To accurately characterize the evolutionary status of the population, a two-dimensional state space spanned by the iteration progress and population diversity is constructed. 

Iteration progress reflects the consumption of temporal resources and is defined as:

(27) $$\xi_{t}=\frac{t}{T_{max}}$$

where t is the current iteration number and $T_{max}$ is the maximum number of iterations. To adapt to the requirements at different stages, $\xi_{t}$ is discretized into three levels:

(28) $$S_{\xi }=\left\{\begin{array}{ll} 1, & 0<\xi_{t}\leq 0.3\\ 2, & 0.3<\xi_{t}\leq 0.7\\ 3, & 0.7<\xi_{t}\leq 1.0 \end{array}\right.$$

These three intervals correspond to the early, middle and late stages of the algorithm’s iteration process, respectively.

Population diversity is a critical indicator of the population’s exploration potential. To eliminate the influence of the scale of the search space, a diversity metric based on normalized standard deviation is adopted:

(29) $$Div_{t}=\frac{1}{D}\sum_{j=1}^{D}\frac{\sigma_{j,t}}{\delta_{j}}$$

where D is the dimensionality; $\sigma_{j,t}$ is the actual standard deviation of the population in the j-th dimension at generation t; and $\delta_{j}$ is the theoretical standard deviation of a uniform distribution in the j-th dimension, calculated as:

(30) $$\delta_{j}=\frac{u_{j}-l_{j}}{\sqrt{12}}$$

where $l_{j}$ and $u_{j}$ represent the lower and upper bounds of the j-th dimension, respectively. Similarly, $Div_{t}$ is discretized into three levels:

(31) $$S_{D}=\left\{\begin{array}{ll} 1, & 0<Div_{t}\leq 0.3\\ 2, & 0.3<Div_{t}\leq 0.7\\ 3, & 0.7<Div_{t}\leq 1.0 \end{array}\right.$$

These three levels correspond to three typical distribution characteristics of the population: highly aggregated, transitional, and highly dispersed.

#### 3.3.2. Action Space

The action space is defined to intelligently regulate the algorithm’s macroscopic search behavior. Q-learning selects one of the following actions based on the current state, instead of the random selection in the original algorithm:

(1) Action 1 (Exploration): Execute the adaptive dimension selection strategy described in Section 3 for position updates. This action aims to maintain population diversity and prevent premature convergence in complex multi-modal landscapes.
(2) Action 2 (Exploitation): Execute the starfish predation strategy described in Section 2. This action focuses on the precise exploitation of discovered high-quality regions to enhance convergence precision.

Based on the definitions above, a Q-table with dimensions of 3×3×2 is constructed. This design covers nine typical evolutionary states, ensuring sufficient update frequency for each state-action pair to facilitate reliable convergence within limited iterations.

#### 3.3.3. Reward Mechanism

The reward function guides the learning process of Q-learning. To provide effective feedback and enhance convergence stability, a novel dual-mechanism reward function is proposed, which combines rewards for progress with dynamic penalties for stagnation.

Let $f_{best}^{t}$ denote the global best fitness at generation t. If the execution of an action improves the global best fitness, a positive reward is granted. This reward incorporates the change in the population mean fitness $f_{mean}$ to ensure the stability of optimization. Otherwise, a dynamic penalty is applied, which is proportional to the consecutive stagnation count $c_{stag}$. The reward function is defined as:

(32) $$r_{t}=\left\{\begin{array}{ll} 1+log\left(1+\left(f_{best}^{t}-f_{best}^{t+1}\right)\right)+0.1\times log\left(1+max\left(0,\left(f_{mean}^{t}-f_{mean}^{t+1}\right)\right)\right), & f_{best}^{t+1}<f_{best}^{t}\\ -\tau \times c_{stag}, & otherwise \end{array}\right.$$

where τ is the penalty coefficient set to 0.05. The counter $c_{stag}$ increments by 1 during stagnation and resets to 0 upon any successful improvement.

The Q-values are updated according to the Bellman equation:

(33) $$Q\left(s_{t},a_{t}\right)\leftarrow Q\left(s_{t},a_{t}\right)+\alpha \left[r_{t}+\gamma \underset{a^{\prime }}{\max}Q\left(s_{t+1},a^{\prime }\right)-Q\left(s_{t},a_{t}\right)\right]$$

where α is the learning rate and γ is the discount factor. An ε-greedy strategy is employed for action selection. The exploration rate ε linearly decays from 0.5 to 0.01 over the iterations, facilitating a smooth transition from stochastic exploration to deterministic exploitation.

To prevent the algorithm from being trapped in local optima, a rebound mechanism is introduced. When the consecutive stagnation count $c_{stag}$ reaches 5, the exploration rate is updated as $\varepsilon =\min\left(0.9,\varepsilon +0.5\right)$, and $c_{stag}$ is reset to 0. This mechanism effectively revitalizes the global search capability, thereby assisting the algorithm in escaping local optima.

At the initialization phase of the algorithm, all Q-values are initialized to 0. During the continuous interaction between the agent and the environment, these values are dynamically updated. The detailed procedure of the Q-learning strategy is summarized as pseudocode in Algorithm 1.
**Algorithm 1:** Q-learning Strategy in QMSFOA**Input:** Current iteration t, maximum iterations $T_{max}$, Q-learning parameters α, γ and ε, penalty coefficient τ.**Output:** Selected action $a_{t}$.1:    Initialize the Q-table as a 3×3×2 zero matrix.2:    Calculate iteration progress $\xi_{t}$ using Equation (27) and discretize into 3 levels.3:    Calculate population diversity $Div_{t}$ using Equation (29) and discretize into 3 levels.4:    **if** $f_{best}^{t+1}<f_{best}^{t}$5:        Calculate reward using Equation (32).6:        Reset stagnation counter: $c_{stag}\;=\;0$.7:    **else**8:        Increment stagnation counter: $c_{stag}=c_{stag}+1$.9:        Calculate penalty: $r_{t}=-\tau \times c_{stag}$.10:  **end if**11:  Update the Q-value using Equation (33).12:  Decay exploration rate: ε.13:  **if** $c_{stag}\geq 5$14:      Increase exploration rate: $\varepsilon =\min\left(0.9,\varepsilon +0.5\right).$15:      Reset stagnation counter: $c_{stag}\;=\;0$.16:  **end if**17:  Generate a random number $rand\in \left[0,1\right]$.18:  **if** rand < ε19:      Select action $a_{t}$ randomly from action space.20:  **else**21:      Select action $a_{t}$ greedily based on the Q-table.22:  **end if**23:  Store current state and action for the next iteration: $s_{t}\leftarrow s_{t+1}$, $a_{t}\leftarrow a_{t+1}$.24:  **Return** $a_{t}$.

### 3.4. Adaptive T-Distribution Elite Perturbation Strategy

To further exploit potential high-quality regions near the global optimum and prevent stagnation in local optima during the later evolutionary stages, QMSFOA incorporates an adaptive t-distribution elite perturbation strategy at the end of each generation.

**Figure 5:**
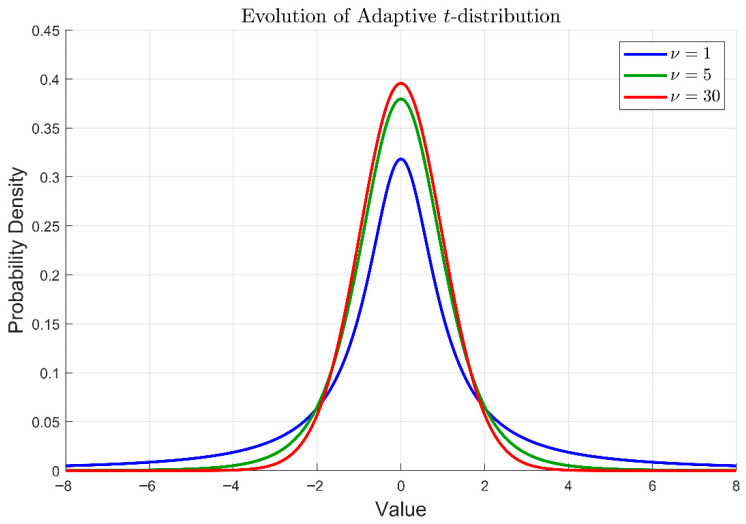
Evolution of adaptive t-distribution.

As a typical parameterized distribution, the profile of the t-distribution is governed by its degree of freedom (DOF) ν. Its probability density function is defined as:

(34) $$f\left(x\right)=\frac{\Gamma \left(\frac{\nu +1}{2}\right)}{\sqrt{\nu \pi }\Gamma \left(\frac{\nu }{2}\right)}{\left(1+\frac{x^{2}}{\nu }\right)}^{-\frac{\nu +1}{2}}$$

where Γ(·) denotes the Gamma function. As ν→1, the t-distribution degrades to the Cauchy distribution, facilitating global exploration. As ν→∞, it asymptotically approaches the Gaussian distribution, favoring precise local exploitation. Figure 5 illustrates the t-distribution under different degrees of freedom.

As shown in Figure 5, with the increase in ν, the distribution curve exhibits thinner tails and a sharper peak. To exploit this characteristic, a time-varying DOF $\nu_{t}$ that increases linearly with the iterative process is designed:

(35) $$\nu_{t}=1+29\cdot \left(\frac{t}{T_{max}}\right)$$

where t and $T_{max}$ represent the current and maximum iteration numbers, respectively. In the early stage, $\nu_{t}$ approaches 1, and the distribution exhibits Cauchy-like characteristics. As iterations proceed, $\nu_{t}$ linearly increases toward 30, causing the distribution to exhibit Gaussian-like characteristics.

Based on the dynamic $\nu_{t}$, the algorithm perturbs the current global best individual $X_{best}^{t}$ to generate a new candidate solution $X_{new}$. The mathematical model is defined as:

(36) $$X_{new}=X_{best}^{t}+\omega \cdot \lambda_{t}\cdot T\left(\nu_{t}\right)$$

where $T\left(\nu_{t}\right)$ represents a random vector following the t-distribution with DOF $\nu_{t}$; ω is the base perturbation coefficient; and $\lambda_{t}$ denotes a dynamic step size factor that decays exponentially with iterations, calculated as:

(37) $$\lambda_{t}=\exp\left(-\frac{t}{T_{max}}\right)$$

If the perturbed individual $X_{new}$ yields a superior fitness value, it replaces the current global best solution. This mechanism significantly enhances the convergence precision of the final solution without disrupting the overall evolutionary trajectory of the population.

The steps of the proposed QMSFOA are presented as pseudo-code in Algorithm 2.
**Algorithm 2:** Pseudo-code of QMSFOA**Input:** Population size N, maximum number of iterations $T_{max}$, dimension D, boundaries $\left[lb,\;ub\right]$, objective function f. **Output:** Global optimal solution $x_{best}$ and its fitness $f_{best}$.1:    Generate initial population using Sobol sequence and ROBL.2:    Initialize global best solution $x_{best}$ and its fitness $f_{best}$.3:    Initialize Q-learning parameters and DE parameters.4:    **for**
t = 1 to $T_{max}$5:        Select the current action $a_{t}$ using Q-learning.6:        **if** $a_{t}==\mathrm{Exploration}$7:            **if** D > 58:                Generate CR for each individual using Equation (16).9:                Determine mutated dimension count using Equation (17).10:              Update individuals using Equation (2).11:              Record successful CR values into $S_{CR}$.12:          **else**13:              Update individuals using Equation (5).14:          **end if**15:      **else if** $a_{t}==\mathrm{Exploitation}$16:          Update individuals using Equation (9).17:      **end if**18:      Evaluate the updated population and update $x_{best}$.19:      Generate scaling factor F for the 2 worst individuals using Equation (20).20:      Select mutation strategy from the strategy pool.21:      Execute selected strategy to generate a candidate solution.22:      Replace the worst individual if the candidate solution is better.23:      Record successful F values into $S_{F}$.24:      **if** t mod LP==025:          Update $\mu_{CR}$ using Equation (18) and $\mu_{F}$ using Equation (22).26:          Update the selection probabilities of the mutation strategies.27:      **end if**28:      Generate perturbed solution for the global best individual using Equation (36).29:      Replace the global best if the perturbed solution is better.30:  **end for**31:  **Return** $x_{best}$ and $f_{best}$.

## 4. Aeromagnetic Path Planning Modeling

In standard geophysical exploration workflows, high-precision environmental data is typically acquired using Digital Surface Models (DSMs), which capture the 3D geometries of non-ground features [46,47]. To simulate realistic aeromagnetic survey scenarios for algorithm verification, this section focuses on two core modeling tasks: (1) constructing a mathematical model of complex 3D environments that mimics the characteristics of DSMs; (2) formulating a comprehensive cost function with strict constraints to generate a reference trajectory satisfying mission requirements.

### 4.1. Complex Mountain Terrain Modeling

Natural mountainous terrains exhibit significant randomness and self-similarity [48,49,50]. To construct a 3D simulation environment that captures both macroscopic geomorphological features and microscopic textural details, a hybrid modeling approach combining multi-frequency fractal noise with Gaussian peaks is adopted.

To balance computational efficiency with geomorphological realism, this study draws upon the fractal superposition concept proposed by Perlin and adopts the method of generating terrain based on spline-interpolated value noise [51].

The base terrain $Z_{b}$ is generated by superimposing M layers of continuous noise functions with varying frequencies and amplitudes, commonly referred to as octaves. Its mathematical model is defined as:

(38) $$Z_{b}\left(x,y\right)=\sum_{k=1}^{M}A_{k}\cdot noise\left(f_{k}x,f_{k}y\right)$$

where $noise\left(\cdot \right)$ denotes a random noise function smoothed via spline interpolation. $A_{k}$ and $f_{k}$ represent the amplitude and frequency of the k-th octave, respectively, following the recurrence relations:

(39) $$A_{k+1}=A_{k}\cdot \rho ,\;0<\rho <1$$

(40) $$f_{k+1}=f_{k}\cdot \tau ,\;\tau >1$$

where ρ represents the persistence, which controls the terrain’s roughness, while τ denotes the lacunarity, which governs the density of textural details.

To simulate steep peaks in specific regions, several Gaussian peaks are superimposed onto the fractal base. Let $\left(x_{ci},y_{ci}\right)$ be the center coordinates of the i-th main peak, $h_{i}$ be the peak height, and $a_{i}$ and $b_{i}$ be the scale parameters along the x and y axes, respectively. The final synthetic terrain height $Z_{t}\left(x,y\right)$ is expressed as:

(41) $$Z_{t}\left(x,y\right)=Z_{b}\left(x,y\right)+\sum_{i=1}^{K}h_{i}\exp\left[-\frac{{\left(x-x_{ci}\right)}^{2}}{a_{i}^{2}}-\frac{{\left(y-y_{ci}\right)}^{2}}{b_{i}^{2}}\right]$$

where K is the total number of superimposed Gaussian peaks.

To simulate natural lakes or plain areas and satisfy the minimum altitude constraints for UAV flight, a clamping mechanism is applied. A water level threshold $H_{w}$ is set, where any height below this threshold is rectified to a flat surface. The final 3D simulation terrain model $Z_{f}\left(x,y\right)$ is defined as:

(42) $$Z_{f}\left(x,y\right)=\max\left(Z_{t}\left(x,y\right),H_{w}\right)$$

### 4.2. Obstacle Modeling

To model the non-ground features captured by DSMs in the 3D simulation environment, obstacles encountered in low-altitude aeromagnetic surveys are categorized into two types [52]: (1) columnar obstacles, such as vegetation and transmission towers; (2) linear obstacles, such as suspended high-voltage power lines. Based on their respective physical geometries, cylindrical bounding box models and spatial line segment models are constructed.

#### 4.2.1. Vegetation Obstacle Model

Considering the stochastic distribution of trees in mountainous terrain and their obstruction to low-altitude flight, individual trees are modeled as vertical cylinders. Assuming there are N trees within the simulation area, the spatial position of the i-th tree is determined by its root coordinates $\left(x_{i},y_{i}\right)$ and the corresponding terrain elevation $Z_{f}\left(x_{i},y_{i}\right)$. The spatial set $\Omega_{tree}^{i}$ occupied by the tree is defined as:

(43) $$\Omega_{tree}^{i}=\left\{\left(x,y,z\right)|{\left(x-x_{i}\right)}^{2}+{\left(y-y_{i}\right)}^{2}\leq R_{i}^{2},\;Z_{f}\leq z\leq Z_{f}+H_{i}\right\}$$

where $H_{i}$ is the tree height and $R_{i}$ is the crown radius. In path planning, the union of all vegetation regions constitutes an untraversable hard constraint space.

#### 4.2.2. Power Transmission Infrastructure Model

The power transmission infrastructure, comprising towers and suspended cables, represents a critical threat in low-altitude aeromagnetic surveys. Due to the extremely small diameter of cables, traditional occupancy grid maps struggle to capture these sparse structures accurately. As a result, a component-based geometric modeling approach is adopted.

Transmission towers possess fixed physical structures and are modeled as vertical cylinders. Similar to the vegetation model, let the k-th tower be located at $\left(x_{k},y_{k}\right)$. Its geometric definition is:

(44) $$\Omega_{tower}^{k}=\left\{\left(x,y,z\right)|{\left(x-x_{k}\right)}^{2}+{\left(y-y_{k}\right)}^{2}\leq R_{k}^{2},\;Z_{f}\leq z\leq Z_{f}+H_{k}\right\}$$

where $R_{k}$ is the minimal bounding cylinder radius of the tower, and $H_{k}$ is the total height.

Suspended cables connect the hanging points $P_{A}$ and $P_{B}$ of two adjacent towers. They are modeled as finite-length line segments in 3D space, denoted as $L_{obs}=P_{A}P_{B}$. To precisely quantify the collision risk, it is necessary to calculate the shortest Euclidean distance between the current UAV flight segment $L_{uav}$ and the obstacle cable $L_{obs}$.

Let the current UAV flight segment connect waypoints $P_{i}$ and $P_{i+1}$. Parametric equations are formulated for both the obstacle cable and the flight segment. Let $Q\left(v\right)$ be an arbitrary point on the cable and $P\left(u\right)$ be an arbitrary point on the flight segment, defined as:

(45) $$\left\{\begin{array}{ll} Q\left(v\right)=P_{A}+v\cdot \left(P_{B}-P_{A}\right), & 0\leq v\leq 1\\ P\left(u\right)=P_{i}+u\cdot \left(P_{i+1}-P_{i}\right), & 0\leq u\leq 1 \end{array}\right.$$

where v and u are the normalized path parameters for the cable and the flight segment, respectively.

In this case, finding the shortest distance $d_{min}$ between the segment and the cable is transformed into an optimization problem of calculating the minimum distance between two spatial skew line segments. The mathematical model is defined as:

(46) $$d_{min}=\underset{0\leq u,v\leq 1}{\min}\left\|P\left(u\right)-Q\left(v\right)\right\|$$

A geometric method is utilized to directly solve this equation to obtain the minimum clearance $d_{min}$ between the UAV and the power lines.

### 4.3. Comprehensive Objective Function

Path planning represents a multi-constrained optimization problem. To address the competing mission requirements of low-altitude aeromagnetic surveys, this study constructs a comprehensive objective function incorporating five critical components: path length, flight altitude, collision threat, attitude smoothness, and course holding. In multi-objective optimization, commonly used approaches include Pareto optimization and the weighted sum method. Although Pareto optimization can reveal trade-offs among multiple objectives, its computational complexity increases with the number of objectives, and the resulting high-dimensional Pareto front is difficult to visualize and utilize in practice [53,54]. Considering that aeromagnetic surveys typically require a single executable optimal flight trajectory, the weighted sum method is adopted in this study.

The total objective function F is formulated as the linear weighted sum of these individual costs:

(47) $$F=\sum_{i=1}^{5}\omega_{i}F_{i}$$

where $\omega_{i}$ denotes the weight coefficient of the i-th cost component, and $F_{1}$ through $F_{5}$ correspond to the five sub-costs mentioned above.

#### 4.3.1. Path Length Cost

The path length is directly related to the UAV’s energy consumption. Reducing the total path length extends the effective survey duration within the constraints of limited battery capacity. The path length cost $F_{1}$ is defined as the cumulative Euclidean distance between adjacent waypoints:

(48) $$F_{1}=\sum_{i=1}^{N-1}\left\|P_{i+1}-P_{i}\right\|=\sum_{i=1}^{N-1}\sqrt{{\left(x_{i+1}-x_{i}\right)}^{2}+{\left(y_{i+1}-y_{i}\right)}^{2}+{\left(z_{i+1}-z_{i}\right)}^{2}}$$

where $P_{i}\left(x_{i},y_{i}{,z}_{i}\right)$ denotes the spatial coordinates of the i-th waypoint, and N is the total number of waypoints.

#### 4.3.2. Flight Altitude Cost

**Figure 6:**
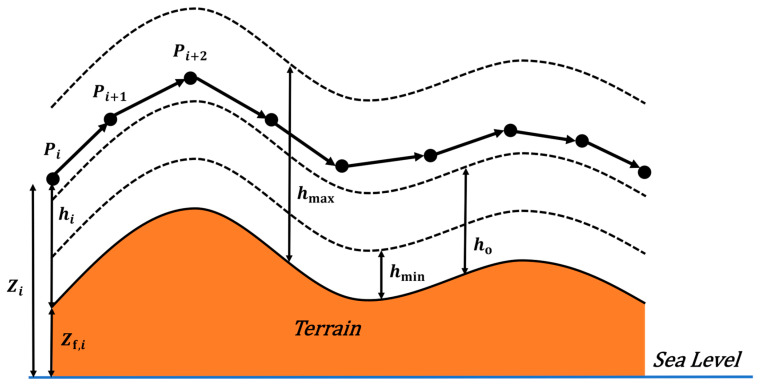
The altitude cost model.

Flight altitude is a critical constraint in aeromagnetic surveys. The UAV should operate at low altitude to improve magnetic anomaly resolution. At the same time, it must maintain minimum terrain clearance and avoid abrupt altitude variations that may trigger maneuver-induced magnetic noise. Figure 6 illustrates the altitude cost model.

Let $Z_{i}$ be the absolute altitude of the i-th waypoint and $Z_{f,i}$ be the corresponding terrain elevation. The ground clearance is defined as $h_{i}=Z_{i}-Z_{f,i}$. Let $h_{o}$ denote the optimal detection clearance, and the interval $\left[h_{min},h_{max}\right]$ represent the safe flight corridor. Based on this model, a composite cost function comprising detection resolution cost $J_{1}$, safety interval cost $J_{2}$, and smoothness cost $J_{3}$ is constructed.

The detection resolution cost $J_{1}$ penalizes deviations above the optimal detection clearance $h_{o}$, formulated as:

(49) $$J_{1}=\sum_{i=1}^{N}j_{1}\left(h_{i}\right),\;j_{1}\left(h_{i}\right)=\left\{\begin{array}{cc} h_{i}-h_{o}, & h_{i}>h_{o}\\ 0, & h_{i}\leq h_{o} \end{array}\right.$$

The safety interval cost $J_{2}$ imposes penalties for violating the safe flight corridor, formulated as:

(50) $$J_{2}=\sum_{i=1}^{N}j_{2}\left(h_{i}\right),\;j_{2}\left(h_{i}\right)=\left\{\begin{array}{cc} \infty , & h_{i}\leq 0\\ h_{min}-h_{i}, & 0<h_{i}<h_{min}\\ 0, & h_{min}\leq h_{i}\leq h_{max}\\ h_{i}-h_{max}, & h_{i}>h_{max} \end{array}\right.$$

The smoothness cost $J_{3}$ constrains the vertical acceleration by calculating the squared second-order difference of altitude across consecutive waypoints:

(51) $$J_{3}=\sum_{i=1}^{N-2}{\left(\left(Z_{i+2}-Z_{i+1}\right)-\left(Z_{i+1}-Z_{i}\right)\right)}^{2}$$

The total flight altitude cost $F_{2}$ is constructed as the weighted sum of these three components:

(52) $$F_{2}=\alpha_{1}J_{1}+\alpha_{2}J_{2}+\alpha_{3}J_{3}$$

where $\alpha_{1}$, $\alpha_{2}$, and $\alpha_{3}$ are the specific weighting coefficients.

#### 4.3.3. Collision Threat Cost

**Figure 7:**
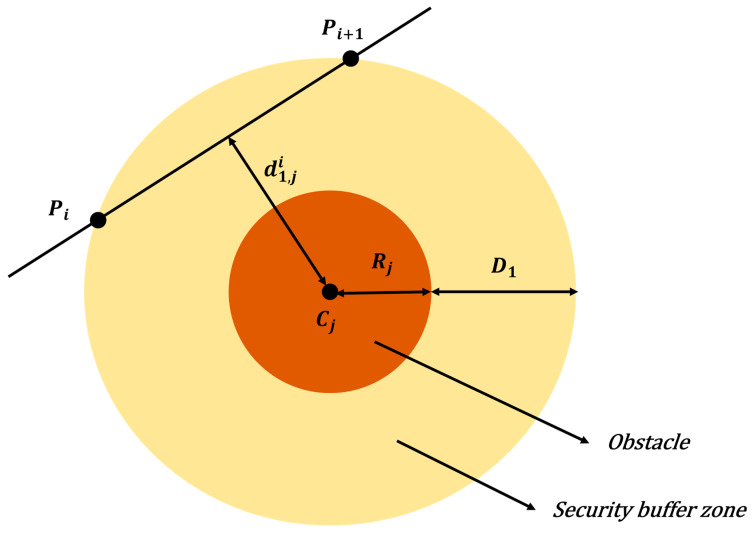
The cost model for cylindrical obstacles.

During aeromagnetic surveys, UAVs encounter various obstacles, such as trees and high-voltage power lines, which pose potential collision hazards. A collision threat cost is introduced to guide the optimization algorithm toward collision-free trajectories, thereby ensuring flight safety. The cost model for cylindrical obstacles is illustrated in Figure 7.

Let the j-th cylindrical obstacle be defined by a base center $C_{j}$ and a radius $R_{j}$. Let $d_{1,j}^{i}$ denote the horizontal distance from the path segment $P_{i}P_{i+1}$ to the cylinder’s central axis. The threat cost function $T_{1,j}^{i}$ is defined as:

(53) $$T_{1,j}^{i}=\left\{\begin{array}{ll} 0, & d_{1,j}^{i}\geq D_{1}+R_{j}\\ \gamma_{1}\cdot \left(D_{1}+R_{j}-d_{1,j}^{i}\right), & R_{j}<d_{1,j}^{i}<D_{1}+R_{j}\\ \infty , & d_{1,j}^{i}\leq R_{j} \end{array}\right.$$

where $\gamma_{1}$ is the weighting coefficient for cylindrical threats, and $D_{1}$ represents the safety buffer distance.

**Figure 8:**
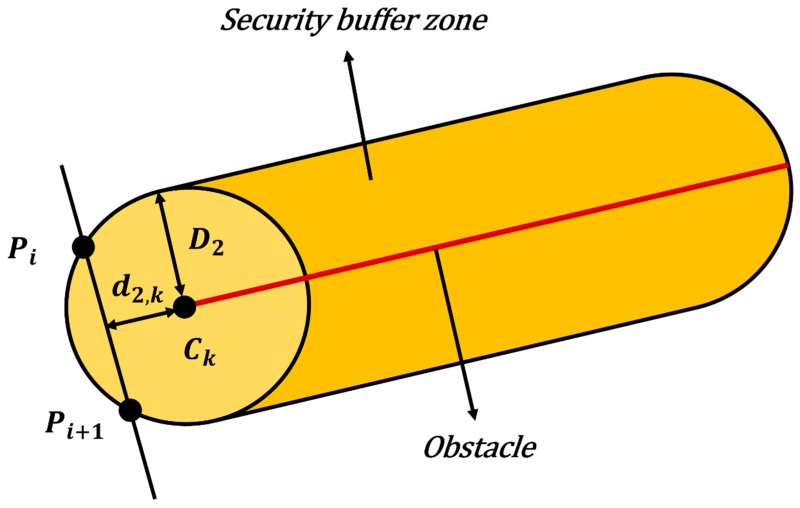
The cost model for linear obstacles.

The cost model for linear obstacles is illustrated in Figure 8.

Let $d_{2,k}^{i}$ denote the shortest Euclidean distance from the path segment $P_{i}P_{i+1}$ to the k-th cable. The threat cost function $T_{2,k}^{i}$ is defined as:

(54) $$T_{2,k}^{i}=\left\{\begin{array}{ll} 0, & d_{2,k}^{i}\geq D_{2}\\ \gamma_{2}\cdot \left(D_{2}-d_{2,k}^{i}\right), & 0<d_{2,k}^{i}<D_{2}\\ \infty , & d_{2,k}^{i}\leq 0 \end{array}\right.$$

where $\gamma_{2}$ is the weighting coefficient for linear threats, and $D_{2}$ is the safety buffer distance.

The total collision threat cost $F_{3}$ is the sum of risks from all obstacles:

(55) $$F_{3}=\sum_{i=1}^{N-1}\left(\sum_{j=1}^{J}T_{1,j}^{i}+\sum_{k=1}^{K}T_{2,k}^{i}\right)$$

where N is the number of waypoints, and J and K represent the total number of cylindrical and linear obstacles, respectively.

#### 4.3.4. Attitude Smoothness Cost

**Figure 9:**
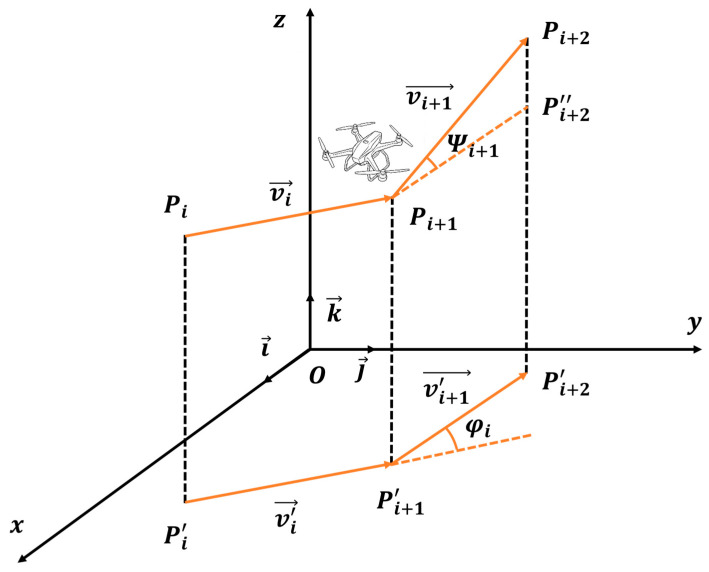
The smoothness cost model.

High-precision magnetometers are extremely sensitive to variations in carrier attitude. Abrupt turns or climbs trigger maneuver-induced magnetic noise, severely degrading the signal-to-noise ratio (SNR) of the measured data. Therefore, the horizontal turning angle and vertical pitch angle variation of the flight path must be strictly constrained. The smoothness cost model is illustrated in Figure 9.

As shown in Figure 9, let $P_{i}$, $P_{i+1}$, $P_{i+2}$ be three consecutive waypoints. The path segment vector is defined as $\vec{v_{i}}=\vec{P_{i}P_{i+1}}$. Let $\vec{k}$ be the unit vector along the z-axis. The projection of the segment vector onto the horizontal plane, $\vec{v_{i}^{\prime }}$, is calculated as:

(56) $$\vec{v_{i}^{\prime }}=\vec{k}\times \left(\vec{v_{i}}\times \vec{k}\right)$$

Based on the projected vectors of two consecutive path segments, $\vec{v_{i}^{\prime }}$ and $\vec{v_{i+1}^{\prime }}$, the horizontal turning angle $\varphi_{i}$ is calculated as:

(57) $$\varphi_{i}=\arccos\left(\frac{\vec{v_{i}^{\prime }}\cdot \vec{v_{i+1}^{\prime }}}{\left|\vec{v_{i}^{\prime }}\right|\cdot \left|\vec{v_{i+1}^{\prime }}\right|}\right)$$

Similarly, the vertical pitch angle $\Psi_{i}$ is computed for each path segment based on the elevation change between consecutive waypoints:

(58) $$\Psi_{i}=\arctan\left(\frac{z_{i+1}-z_{i}}{\left|\vec{v_{i}^{\prime }}\right|}\right)$$

The attitude smoothness cost $F_{4}$ is defined as the linear weighted sum of the cumulative horizontal turning angle and vertical pitch angle variation:

(59) $$F_{4}=\lambda_{1}\sum_{i=1}^{N-2}\varphi_{i}+\lambda_{2}\sum_{i=1}^{N-2}\left|\Psi_{i+1}-\Psi_{i}\right|$$

where $\lambda_{1}$ and $\lambda_{2}$ are the weighting coefficients for horizontal turning and vertical pitch smoothness, respectively.

#### 4.3.5. Course Holding Cost

**Figure 10:**
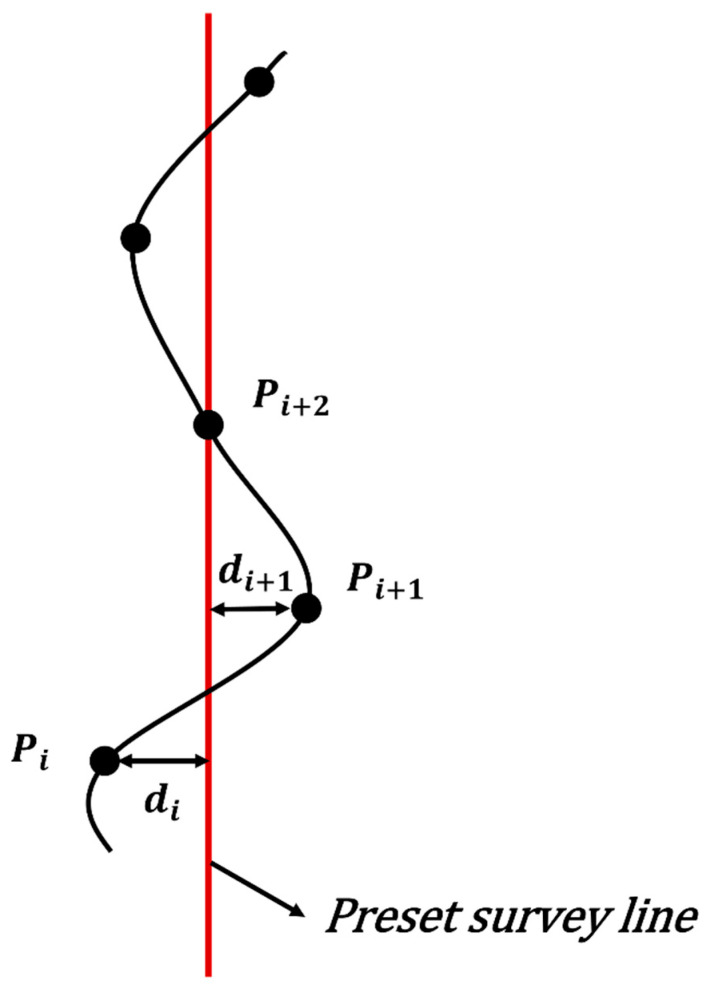
Course holding cost model.

Aeromagnetic surveys require the UAV to closely follow pre-planned survey lines to ensure spatial data consistency and uniform grid coverage. To limit lateral deviation during obstacle avoidance and suppress unnecessary lateral oscillation, a course holding cost is introduced. The course holding cost model is illustrated in Figure 10.

To constrain the macroscopic straightness of the trajectory, a cumulative lateral error term $f_{1}$ is defined. Let $L_{ref}$ be the preset survey line, and $d_{i}$ be the perpendicular distance from the i-th waypoint to the reference line. The calculation of $f_{1}$ is as follows:

(60) $$f_{1}=\sum_{i=1}^{N}d_{i}$$

Frequent serpentine corrections on either side of the survey line introduce significant motion-induced magnetic interference. To mitigate this, a yaw stability term $f_{2}$ is introduced:

(61) $$f_{2}=\sum_{i=1}^{N-1}\left|d_{i+1}-d_{i}\right|$$

Combining these two metrics, the total course holding cost $F_{5}$ is defined as:

(62) $$F_{5}=w_{1}f_{1}+w_{2}f_{2}$$

where $w_{1}$ and $w_{2}$ are the weighting coefficients for the cross-track error term and the yaw stability term, respectively.

### 4.4. Trajectory Smoothing via Cubic B-Splines

The raw path generated by the optimization algorithm consists of a sequence of discrete waypoints. Directly tracking these points induces abrupt turns and attitude changes, violating the UAV kinematic constraints [55]. To address this, cubic B-spline curves [56] are employed to smooth the discrete path.

The method utilizes the optimized discrete waypoints as control points to generate a trajectory with continuous curvature. The mathematical expression of the cubic B-spline curve $S\left(u\right)$ is defined as:

(63) $$S\left(u\right)=\sum_{i=0}^{n}P_{i}\cdot N_{i,k}\left(u\right),\;0\leq u\leq u_{max}$$

where $P_{i}$ represents the control points, k=3 denotes the degree of the B-spline, and $N_{i,k}\left(u\right)$ are the normalized basis functions defined by the De Boor–Cox recursive formula:

(64) $$N_{i,0}\left(u\right)=\left\{\begin{array}{ll} 1, & u_{i}\leq u<u_{i+1}\\ 0, & otherwise \end{array}\right.\;N_{i,k}\left(u\right)=\frac{u-u_{i}}{u_{i+k}-u_{i}}N_{i,k-1}\left(u\right)+\frac{u_{i+k+1}-u}{u_{i+k+1}-u_{i+1}}N_{i+1,k-1}\left(u\right)$$

This smoothing mechanism ensures continuous variations in the UAV’s velocity and acceleration. It effectively satisfies the practical flight requirements for stable aeromagnetic data acquisition.

## 5. Algorithm Comparison Experiments

To ensure experimental objectivity, all simulations were conducted on a 64-bit Windows 11 operating system, equipped with an Intel Core i9-13900H CPU, NVIDIA RTX 4060 GPU, and 16GB RAM. Matlab R2023b served as the simulation software for the experiments.

### 5.1. Experimental Design

To comprehensively evaluate the optimization performance of the proposed QMSFOA, seven representative metaheuristic algorithms were selected as benchmarks: Particle Swarm Optimization (PSO) [24], Grey Wolf Optimizer (GWO) [27], Dung Beetle Optimizer (DBO) [31], Black-winged Kite Algorithm (BKA) [33], Enhanced Dung Beetle Optimizer (EDBO) [37], Enhanced Starfish Optimization Algorithm (SFOAL) [57], and the standard Starfish Optimization Algorithm (SFOA) [38]. Among these, EDBO is an improved variant of the DBO algorithm, while SFOAL serves as an enhanced version of SFOA.

The CEC-2022 benchmark suite was employed for evaluation. For all algorithms, the population size, maximum number of iterations, and problem dimension were set to 30, 1000, and 20, respectively. Each algorithm was independently run 30 times on each test function. Detailed definitions of the CEC-2022 test functions and the key parameter configurations for each algorithm are provided in Table 1 and Table 2, respectively.

### 5.2. Comparative Analysis of Algorithms

The best value, mean, and standard deviation were selected as the primary evaluation metrics, representing the algorithms’ ultimate optimization potential, overall convergence accuracy, and robustness, respectively.

**Figure 11:**
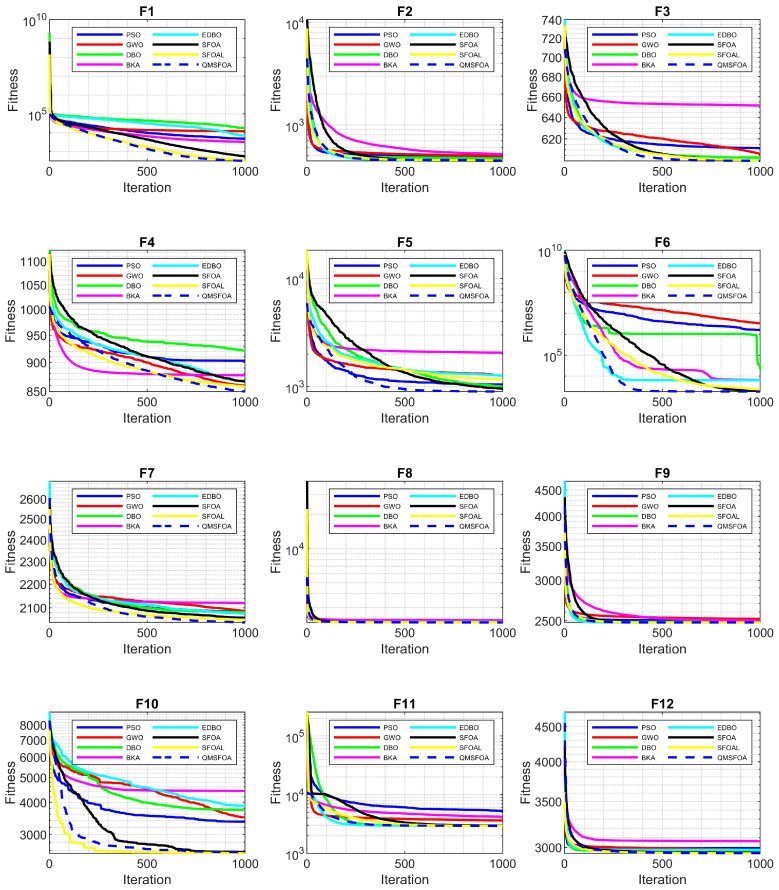
Convergence curves of the algorithms.

The comparative results are summarized in Table 3, and the convergence curves are illustrated in Figure 11.

Table 3 and Figure 11 indicate that QMSFOA achieves superior convergence accuracy and stability on most of the CEC-2022 benchmark functions. Specifically, the algorithm exhibits a significant advantage on the unimodal function $f_{1}$ and achieves the best results on multimodal functions $f_{3}$, $f_{4}$, and $f_{5}$. For the challenging hybrid functions $f_{6}$, $f_{7}$, and $f_{8}$, QMSFOA consistently attains the lowest mean fitness values and standard deviations, highlighting its strong capability of handling complex optimization landscapes. Although slightly outperformed by SFOAL on composition functions $f_{10}$ and $f_{12}$, QMSFOA maintains competitive precision and regains the lead on $f_{9}$ and $f_{11}$. Overall, QMSFOA achieves the best performance in 25 out of 36 statistical metrics across the 12 test functions, demonstrating robust global search capability and strong potential for high-dimensional engineering optimization problems.

**Figure 12:**
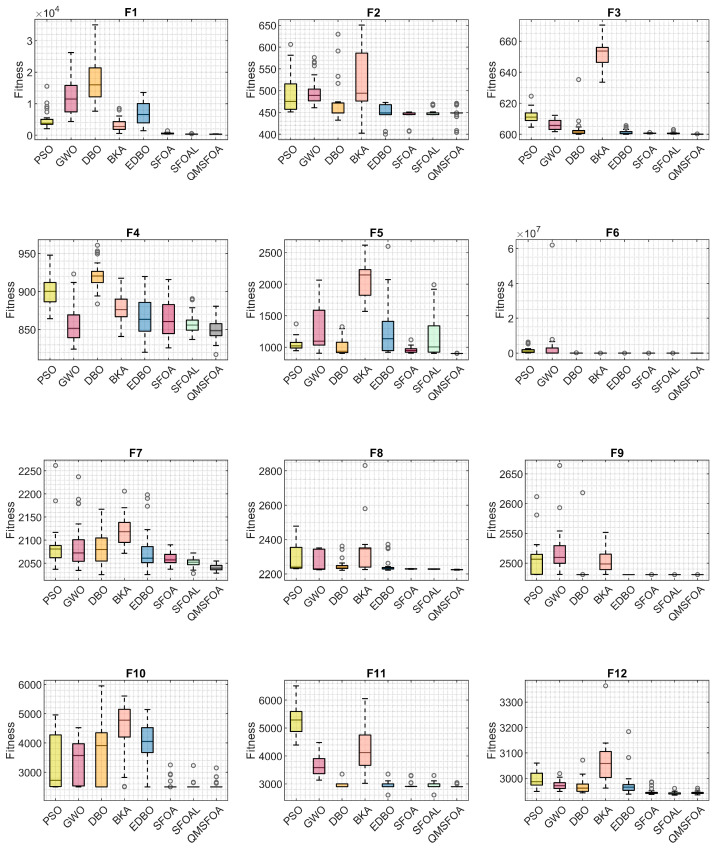
Box plots of the competing algorithms.

To further analyze the distribution characteristics of the solutions, Figure 12 presents the box plots of the competing algorithms.

As illustrated in Figure 12, on functions $f_{1}$, $f_{3}$–$f_{6}$, and $f_{9}$, QMSFOA yields both the lowest minimum values and medians. Notably, on $f_{1}$, $f_{5}$, $f_{6}$, and $f_{12}$, the box plots for QMSFOA are compressed into extremely narrow bands near the best obtained values, indicating low variability and stable performance. For functions $f_{2}$, $f_{7}$, $f_{8}$, $f_{10}$, and $f_{11}$, although QMSFOA does not always yield the best minimum values, its overall box distributions remain highly compact and closely approach the optimal solutions. These observations indicate that QMSFOA possesses a competitive optimization performance and robustness.

### 5.3. Wilcoxon Rank-Sum Test

To statistically assess the significance of the performance differences between QMSFOA and other algorithms, the Wilcoxon rank-sum test was employed. The null hypothesis assumes there is no significant difference between the two compared algorithms. A p-value less than 0.05 leads to the rejection of the null hypothesis, indicating a statistically significant difference, whereas a p-value greater than 0.05 implies no significant difference. Table 4 presents the test results.

Table 4 shows that the p-values for QMSFOA relative to competing algorithms are well below 0.05 on most of the functions, demonstrating that the observed performance differences are statistically significant. Although the difference is less pronounced on a few specific functions, the overall extremely low p-values provide strong evidence for the effectiveness of the multi-strategy improvement framework.

### 5.4. Ablation Experiment

To comprehensively analyze the contributions of the four improvement strategies, an ablation study from two perspectives was conducted. Specifically, the study consists of two parts: (1) incremental analysis: evaluating the performance of SFOA integrated with a single strategy to verify its standalone effectiveness; (2) removal analysis: assessing the performance degradation when omitting each strategy individually from the complete algorithm to demonstrate the importance of each component.

#### 5.4.1. Incremental Analysis

**Figure 13:**
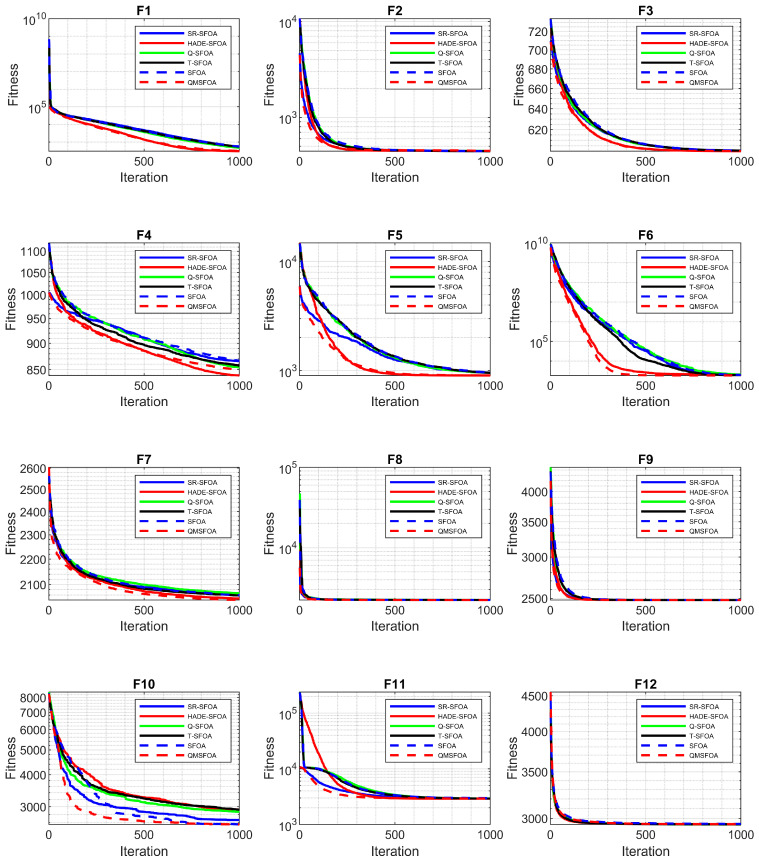
Convergence curves of incremental strategy variants.

To evaluate the contribution of each component, four variant algorithms were constructed by incorporating the following strategies individually into the standard SFOA: Sobol sequence and Refraction Opposition-Based Learning (SR-SFOA), hybrid adaptive differential mutation (HADE-SFOA), Q-learning (Q-SFOA), and adaptive t-distribution perturbation (T-SFOA). These variants were compared with the standard SFOA and the proposed QMSFOA on the CEC-2022 benchmark suite. The results are summarized in Table 5 and the convergence curves are shown in Figure 13.

As shown in Table 5 and Figure 13, SR-SFOA achieves faster convergence in the early iterations by generating a more uniformly distributed initial population. HADE-SFOA serves as the primary contributor to convergence accuracy, exhibiting a superior performance on simpler functions such as $f_{1}$, $f_{3}$, $f_{4}$, and $f_{5}$. This highlights the critical role of the hybrid adaptive differential mutation strategy in accelerating the search process. T-SFOA demonstrates a specialized advantage in escaping local optima, as evidenced by its ability to identify the best solution on the hybrid function $f_{6}$ and the composition function $f_{11}$. Although Q-SFOA exhibits relatively slow convergence on simpler landscapes, it maintains a better balance between exploration and exploitation, outperforming the original SFOA on more complex functions such as $f_{12}$. Overall, QMSFOA achieves the most balanced performance by integrating the complementary strengths of the individual strategies across varying problem complexities.

#### 5.4.2. Removal Analysis

**Figure 14:**
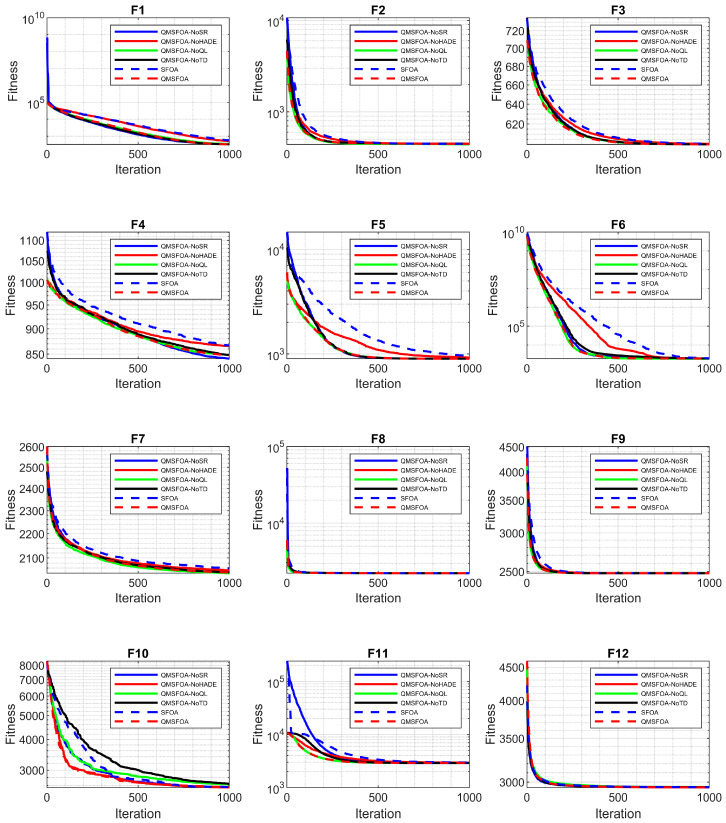
Convergence curves of removal strategy variants.

To further verify the necessity of each component, a removal analysis was conducted. By sequentially removing one strategy from the complete framework, four incomplete variants were evaluated: QMSFOA-NoSR, QMSFOA-NoHADE, QMSFOA-NoQL, and QMSFOA-NoTD. The statistical results and convergence curves are summarized in Table 6 and Figure 14, respectively.

As shown in Table 6 and Figure 14, removing any single strategy leads to performance degradation to varying degrees. When the Sobol sequence and Refraction Opposition-Based Learning are discarded, QMSFOA-NoSR exhibits slower convergence during the early search phases. The removal of the hybrid adaptive differential mutation (QMSFOA-NoHADE) results in a severe loss of convergence accuracy, as evidenced by the significant performance declines on $f_{1}$, $f_{4}$, and $f_{5}$. Furthermore, omitting Q-learning (QMSFOA-NoQL) disrupts the dynamic balance between exploration and exploitation, making the algorithm more susceptible to premature convergence on complex functions such as $f_{10}$. Similarly, without the adaptive t-distribution perturbation, QMSFOA-NoTD shows reduced capability to escape local optima, as reflected in the noticeable performance deterioration on $f_{6}$ and $f_{12}$. These results indicate that each of these four enhancement strategies plays an important role in the search process, collectively contributing to the robust overall optimization performance of QMSFOA.

### 5.5. Computational Complexity Analysis

The computational complexity of the proposed QMSFOA is analyzed to assess its cost and scalability. Let N denote the population size, D the dimensionality of the search space, and $T_{max}$ the maximum number of iterations. The time complexity of the standard SFOA is asymptotically $O\left(T_{max}\times N\times D\right)$. In QMSFOA, the Sobol and ROBL initialization is executed only once with a complexity of $O\left(N\log N+N\times D\right)$, which is negligible compared to the iterative process. During the evolutionary loop, Q-learning introduces operations with complexity $O\left(N\times D\right)$ per iteration. Since the self-adaptive differential mutation acts on only 2 individuals and the t-distribution elite perturbation strategy acts on only 1 individual, these two strategies add merely a linear computational term of $O\left(D\right)$ per iteration. Therefore, combining these components, the dominant computational term remains unchanged. The overall time complexity is maintained at $O\left(T_{max}\times N\times D\right)$.

**Figure 15:**
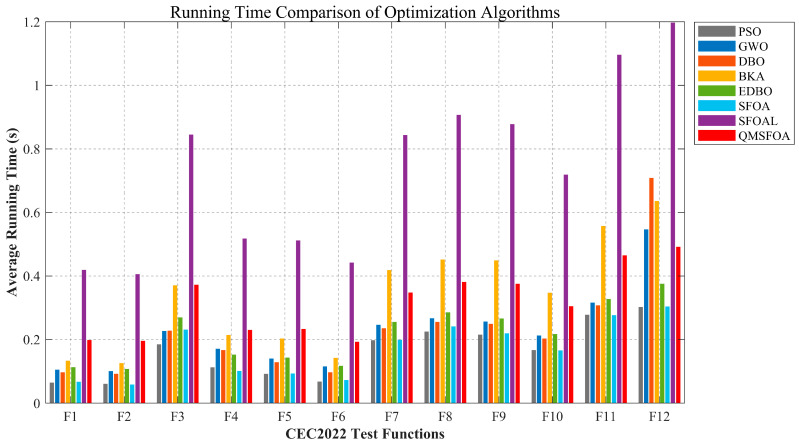
Running time comparison of optimization algorithms.

To evaluate the computational cost in practice, QMSFOA and all competing algorithms were independently run 30 times on the CEC-2022 test suite. The average running time for each function is shown in Figure 15.

Figure 15 shows that although the running time of QMSFOA on most functions is higher than that of algorithms including PSO, GWO, and SFOA, the time gap gradually narrows as the functions become increasingly complex. On certain functions, QMSFOA even outperforms GWO, DBO, and BKA. Notably, the running time of QMSFOA is consistently lower than that of SFOAL. This can be attributed to the fact that SFOAL’s t-distribution mutation and logarithmic spiral opposition-based learning are applied to the entire population, necessitating 3N objective function evaluations per iteration. In addition, consistent with the lightweight design of the standard SFOA, which mutates at most five dimensions during the exploration phase, the proposed QMSFOA proportionally controls the number of mutated dimensions via self-adaptive differential mutation. This avoids the computational redundancy caused by complex mathematical operations across all dimensions, as seen in other algorithms. These results indicate that QMSFOA improves optimization accuracy while effectively balancing computational efficiency.

### 5.6. Parameter Sensitivity Analysis of Q-Learning

**Figure 16:**
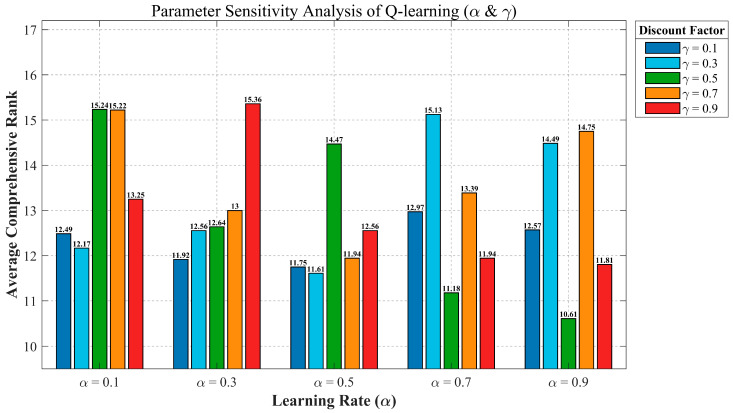
Parameter sensitivity analysis of Q-learning.

In the Q-learning mechanism, the learning rate α and the discount factor γ directly influence the effectiveness of QMSFOA’s dynamic strategy selection. To evaluate the impact of these parameters and identify suitable parameter settings, a sensitivity analysis was conducted on the CEC-2022 test suite using 25 different parameter combinations: α∈{0.1, 0.3, 0.5, 0.7, 0.9} and γ∈{0.1, 0.3, 0.5, 0.7, 0.9}. Figure 16 illustrates the average ranking performance of the algorithm across all test functions under these combinations.

As shown in Figure 16, the performance of the algorithm demonstrates a clear sensitivity to the values of α and γ. QMSFOA achieves the lowest average ranking performance when α=0.9 and γ=0.5, outperforming other parameter combinations. Therefore, α=0.9 and γ=0.5 are adopted as the default parameters for QMSFOA. This configuration contributes to a more balanced and robust optimization performance in various complex tasks.

## 6. Simulation in Aeromagnetic Surveys

### 6.1. Simulation Scenario Setup

**Figure 17:**
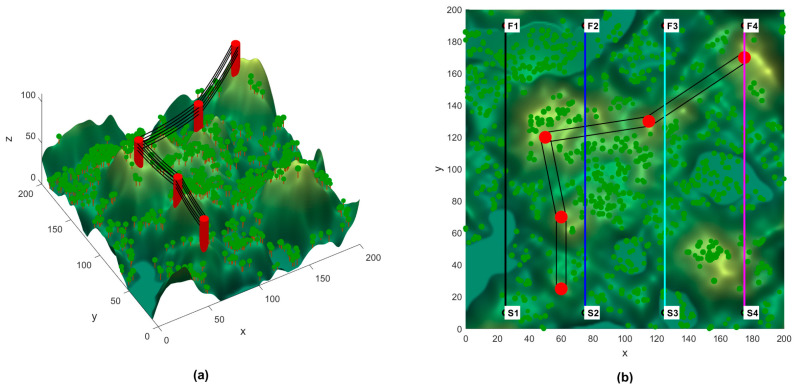
Simulation setup. (**a**) 3D topographical model. (**b**) Preset survey trajectories.

To evaluate QMSFOA’s performance in aeromagnetic surveys, a 3D simulation environment with undulating terrain, mountain peaks, and obstacles was constructed based on the modeling method described in Section 4. The horizontal survey boundaries, defining the target measurement area, were restricted to a 200 × 200 rectangular region. The safe flight corridor $\left[h_{min},h_{max}\right]$ was set to $\left[1,20\right]$, and the optimal detection clearance $h_{o}$ was set to 3. The safety buffer distances for cylindrical and linear obstacles, $D_{1}$ and $D_{2}$, were set to 5. To assess robustness under varying terrain complexities, four survey mission routes with distinct start–goal configurations were designed. The 3D simulation model and the four preset survey lines are shown in Figure 17.

### 6.2. Competing Algorithms and Parameter Settings

The experiments utilized the same six metaheuristic algorithms from the benchmark tests as a comparison group. For all algorithms, the population size was set to 60 and the maximum iterations to 1000. Each algorithm was run independently 30 times under identical conditions.

The weighting coefficients of the objective function were determined according to the dominant physical factors affecting aeromagnetic survey quality. Flight attitude stability directly influences magnetic noise levels, terrain clearance affects signal validity and anomaly resolution, and obstacle avoidance guarantees flight safety. Reflecting these priorities, the weights were set as: $\omega_{1}=0.15$, $\omega_{2}=0.20$, $\omega_{3}=0.20$, $\omega_{4}=0.15$, $\omega_{5}=0.30$.

### 6.3. Analysis of Experimental Results

**Figure 18:**
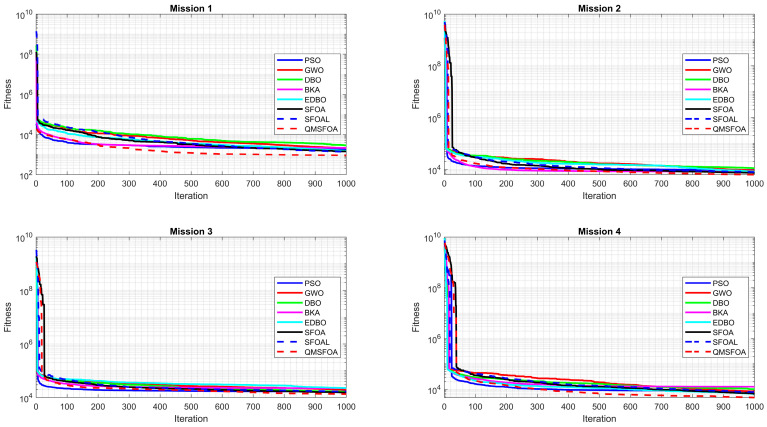
Convergence curves for aeromagnetic path planning.

**Figure 19:**
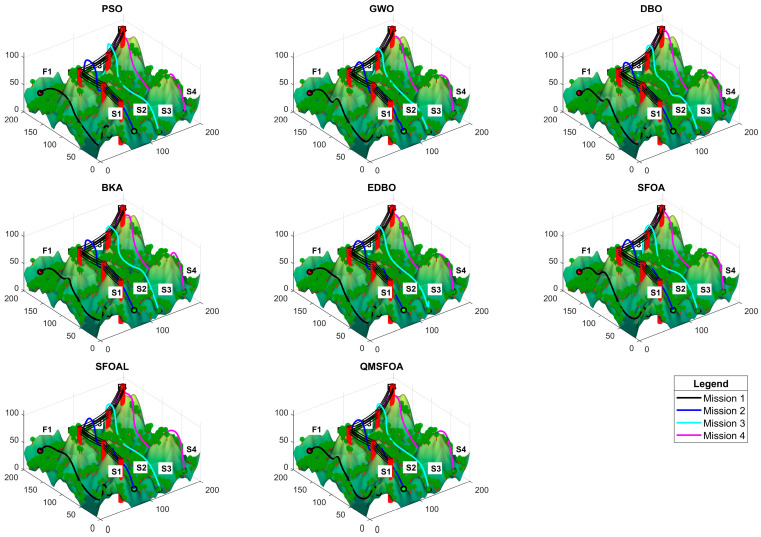
Graphs of 3D trajectories.

**Figure 20:**
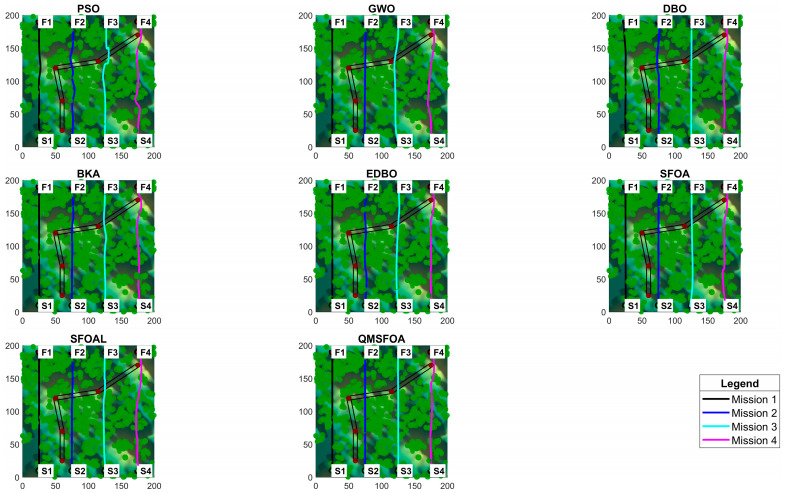
Images of 2D planar trajectories.

**Figure 21:**
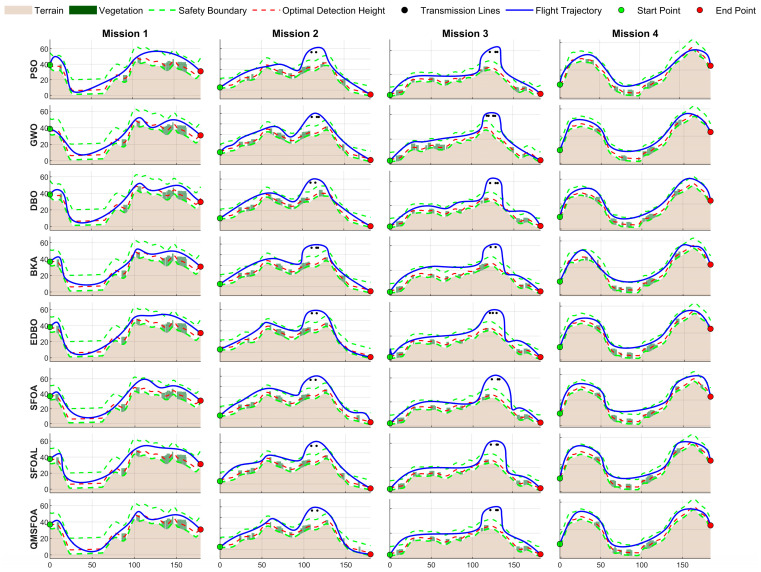
The vertical trajectory profile.

The statistical results of QMSFOA and the six competing algorithms across the four aeromagnetic missions are summarized in Table 7. The convergence curves are shown in Figure 18. The generated 3D trajectories, 2D planar trajectories, and vertical trajectory profile are presented in Figure 19, Figure 20, and Figure 21, respectively.

The results in Table 7 indicate that QMSFOA exhibits consistently superior optimization accuracy and stability. Across all missions, QMSFOA attains the lowest mean fitness values, reflecting its strong global optimization capability. In terms of solution stability, QMSFOA achieves the lowest standard deviations in Missions 1, 2 and 4. Although SFOA and SFOAL obtain slightly lower standard deviations in Mission 3, these differences are marginal. This suggests that QMSFOA maintains a favorable balance between search accuracy and stability. Furthermore, despite BKA and EDBO attaining slightly better best values in Missions 1 and 4, respectively, their standard deviations are significantly higher than those of QMSFOA, suggesting lower performance consistency. Overall, QMSFOA possesses higher reliability for engineering applications.

As illustrated by the convergence curves in Figure 18, QMSFOA shows a steep descent during the early iterations, indicating a rapid convergence rate. In the middle and later stages, while several competing algorithms experience convergence stagnation, QMSFOA continues to reduce the objective value. These results suggest strong global search capability and improved ability to avoid local optima, which is important for aeromagnetic missions conducted in complex terrain environments.

The 3D and 2D trajectory plots in Figure 19 and Figure 20 indicate that while all algorithms achieved obstacle avoidance, marked differences exist in path quality. Compared with the benchmark algorithms, the trajectory generated by QMSFOA exhibits stronger alignment with the preset survey line. While minor deviations may occur due to local obstacle constraints, QMSFOA effectively eliminates the severe sharp turns and repetitive lateral oscillations observed in other methods such as PSO. This smoother heading behavior is expected to mitigate maneuver-induced magnetic interference, which is beneficial for maintaining data quality during aeromagnetic surveys.

The side-profile view in Figure 21 illustrates that QMSFOA achieves a robust balance between obstacle avoidance and terrain following. Except for necessary vertical maneuvers when encountering vegetation and power lines, the trajectory remains close to the optimal detection height for most flight segments while staying within the defined safe flight corridor. This suggests that QMSFOA exhibits strong terrain-following capability, which is crucial for preserving high geomagnetic signal resolution.

## 7. Conclusions

This study addresses the dual challenges of flight safety and data quality in UAV aeromagnetic surveys within complex mountainous environments by proposing a Q-learning-assisted multi-strategy Starfish Optimization Algorithm (QMSFOA). Benchmark experiments on the CEC-2022 test suite show that QMSFOA secured the best performance in 25 of the 36 statistical metrics across 12 test functions, demonstrating superior convergence speed and solution accuracy compared to the selected baseline algorithms. For engineering validation, a 3D path planning framework integrating terrain following, obstacle avoidance, and flight performance constraints was constructed. Simulation results verify the feasibility of the proposed method for aeromagnetic path planning in complex terrain scenarios.

Despite these achievements, several limitations remain. Since QMSFOA is fundamentally a metaheuristic algorithm designed for offline optimization in deterministic scenarios, it has inherent limitations in real-time responsiveness when confronting unforeseen dynamic threats. Furthermore, the current simulations primarily rely on a point-mass kinematic model and lack practical engineering validation, making it difficult to fully reflect the impact of ubiquitous sensor noise, localization uncertainties, and complex aerodynamic disturbances in field operations. Future research will focus on developing a more comprehensive flight control framework. At the algorithmic level, the proposed QMSFOA can serve as an upper-level offline global path planner to generate high-quality reference trajectories, while lower-level local controllers, such as Model Predictive Control (MPC) [58], perform trajectory tracking and real-time corrections. Additionally, to handle unforeseen dynamic threats, Deep Reinforcement Learning (DRL) [59] can be integrated for real-time obstacle avoidance while mitigating maneuver-induced magnetic interference. At the engineering validation level, future work will further advance toward practical deployment by incorporating Hardware-in-the-Loop (HIL) simulations [60] and real-world flight experiments to evaluate the robustness of the proposed method under various practical uncertainties.

As noted in [23], the field of path planning is evolving from single-agent systems toward multi-agent collaboration. In future work, we will explore extending the current centralized framework into a distributed multi-agent system to fulfill the demands of large-scale collaborative operations.

## Figures and Tables

**Table 1:** CEC-2022 test suite.

Name	Type	Function	$f_{min}$
$f_{1}$	**Unimodal functions**	**Shifted and full rotated Zakharov function**	**300**
$f_{2}$	**Basic functions**	**Shifted and rotated Rosenbrock’s function**	**400**
$f_{3}$		**Shifted and rotated expanded Schaffer’s F6 function**	**600**
$f_{4}$		**Shifted and rotated non-continuous Rastrigin’s function**	**800**
$f_{5}$		**Shifted and rotated Levy function**	**900**
$f_{6}$	**Hybrid functions**	**Hybrid function 1 (*n* = 3)**	**1800**
$f_{7}$		**Hybrid function 2 (*n* = 6)**	**2000**
$f_{8}$		**Hybrid function 3 (*n* = 5)**	**2200**
$f_{9}$	**Composition functions**	**Composition function 1 (*n* = 5)**	**2300**
$f_{10}$		**Composition function 2 (*n* = 4)**	**2400**
$f_{11}$		**Composition function 3 (*n* = 5)**	**2600**
$f_{12}$		**Composition function 4 (*n* = 6)**	**2700**

**Table 2:** Key parameter configurations.

Algorithm	Parameters	Values
PSO	Inertia weight	0.8
	Learning factors	2.0, 2.0
GWO	Convergence constant	2.0
DBO	Deflection coefficient	0.1
	Constant parameters	0.3, 0.5
BKA	Attacking probability	0.9
EDBO	MOP control parameter	0.3
	Adjustment factor	0.5
	T-distribution scale factor	10
SFOA	Probability parameter	0.5
SFOAL	Spiral shape constant	1.0
QMSFOA	Learning rate (α)	0.9
	Discount factor (γ)	0.5
	Initial exploration rate (ε)	0.9
	Learning Period (LP)	25
	Initial Mean CR ($\mu_{CR}$)	0.5
	Initial Mean F ($\mu_{F}$)	0.5

**Table 3:** Experimental results on CEC-2022 test functions (Dim = 20).

Functions	Metrics	PSO	GWO	DBO	BKA	EDBO	SFOA	SFOAL	QMSFOA
$f_{1}$	Best	2.0625 × 10^3^	4.3476 × 10^3^	7.5794 × 10^3^	5.3897 × 10^2^	1.4101 × 10^3^	3.4273 × 10^2^	3.0723 × 10^2^	**3.0039 × 10^2^**
	Mean	4.8084 × 10^3^	1.2070 × 10^4^	1.7685 × 10^4^	3.2453 × 10^3^	6.9524 × 10^3^	5.6335 × 10^2^	3.4947 × 10^2^	**3.2321 × 10^2^**
	SD	2.8192 × 10^3^	5.2906 × 10^3^	7.0532 × 10^3^	2.0132 × 10^3^	3.4529 × 10^3^	2.2323 × 10^2^	4.8140 × 10^1^	**2.8478 × 10^1^**
$f_{2}$	Best	4.5122 × 10^2^	4.6089 × 10^2^	4.3226 × 10^2^	4.0231 × 10^2^	**4.0004 × 10^2^**	4.0682 × 10^2^	4.4490 × 10^2^	4.0424 × 10^2^
	Mean	4.9378 × 10^2^	4.9825 × 10^2^	4.7087 × 10^2^	5.1831 × 10^2^	4.4847 × 10^2^	**4.4548 × 10^2^**	4.5064 × 10^2^	4.4796 × 10^2^
	SD	4.5410 × 10^1^	3.2658 × 10^1^	4.3837 × 10^1^	6.5452 × 10^1^	2.0334 × 10^1^	1.0510 × 10^1^	**7.2457**	1.6619 × 10^1^
$f_{3}$	Best	6.0464 × 10^2^	6.0167 × 10^2^	**6.0005 × 10^2^**	6.3553 × 10^2^	6.004 × 10^2^	6.0026 × 10^2^	6.0013 × 10^2^	**6.0003 × 10^2^**
	Mean	6.1167 × 10^2^	6.0620 × 10^2^	6.0299 × 10^2^	6.5167 × 10^2^	6.0135 × 10^2^	6.0066 × 10^2^	6.0067 × 10^2^	**6.0010 × 10^2^**
	SD	3.9033	3.1572	6.3840	8.6477	1.5251	2.2680 × 10^−1^	6.9380 × 10^−1^	**5.2000 × 10^−2^**
$f_{4}$	Best	8.6425 × 10^2^	8.2415 × 10^2^	8.8359 × 10^2^	8.4081 × 10^2^	8.1990 × 10^2^	8.2590 × 10^2^	8.3689 × 10^2^	**8.1711 × 10^2^**
	Mean	9.0305 × 10^2^	8.5975 × 10^2^	9.2080 × 10^2^	8.7721 × 10^2^	8.6719 × 10^2^	8.6646 × 10^2^	8.5783 × 10^2^	**8.4956 × 10^2^**
	SD	2.1708 × 10^1^	2.6489 × 10^1^	1.7517 × 10^1^	1.7771 × 10^1^	2.3518 × 10^1^	2.5146 × 10^1^	**1.2976 × 10^1^**	1.3741 × 10^1^
$f_{5}$	Best	9.4595 × 10^2^	9.0529 × 10^2^	9.0312 × 10^2^	1.5686 × 10^3^	9.2135 × 10^2^	9.0636 × 10^2^	9.0242× 10^2^	**9.0000 × 10^2^**
	Mean	1.0469 × 10^3^	1.2619 × 10^3^	1.0019 × 10^3^	2.0615 × 10^3^	1.2627× 10^3^	9.5761 × 10^2^	1.1527 × 10^3^	**9.0041 × 10^2^**
	SD	8.9344 × 10^1^	3.2204 × 10^2^	1.1208 × 10^2^	2.6593 × 10^2^	4.0021 × 10^2^	4.7663 × 10^1^	3.0569 × 10^2^	**1.3514**
$f_{6}$	Best	1.2831 × 10^5^	2.4611 × 10^3^	2.0968 × 10^3^	2.1073 × 10^3^	1.9389× 10^3^	1.8764 × 10^3^	1.8614 × 10^3^	**1.8131 × 10^3^**
	Mean	1.5879 × 10^6^	3.3380 × 10^6^	2.1638 × 10^4^	6.5419 × 10^3^	6.4011× 10^3^	1.9687 × 10^3^	2.2577 × 10^3^	**1.8475 × 10^3^**
	SD	1.6243 × 10^6^	1.1288 × 10^7^	3.3798 × 10^4^	5.7847 × 10^3^	6.8777 × 10^3^	7.8783 × 10^1^	1.2184 × 10^3^	**2.2631 × 10^1^**
$f_{7}$	Best	2.0370 × 10^3^	2.0343 × 10^3^	**2.0253 × 10^3^**	2.0713 × 10^3^	2.0255 × 10^3^	2.0372 × 10^3^	2.2078 × 10^3^	2.0284 × 10^3^
	Mean	2.0845 × 10^3^	2.0865 × 10^3^	2.0809 × 10^3^	2.1193 × 10^3^	2.0765 × 10^3^	2.0591 × 10^3^	2.0514 × 10^3^	**2.0407 × 10^3^**
	SD	4.3093 × 10^1^	5.0024 × 10^1^	3.2772 × 10^1^	2.9642 × 10^1^	4.4687 × 10^1^	1.2469 × 10^1^	9.8681	**7.2294**
$f_{8}$	Best	2.2298 × 10^3^	2.2237 × 10^3^	**2.2214 × 10^3^**	2.2254 × 10^3^	2.2219 × 10^3^	2.2265 × 10^3^	2.2261 × 10^3^	2.2220 × 10^3^
	Mean	2.2867 × 10^3^	2.2612 × 10^3^	2.2510 × 10^3^	2.3294 × 10^3^	2.2449 × 10^3^	2.2293 × 10^3^	2.2282 × 10^3^	**2.2247 × 10^3^**
	SD	7.0100 × 10^1^	5.2823 × 10^1^	3.6161 × 10^1^	1.2129 × 10^2^	3.9043 × 10^1^	1.2586	1.1323	**9.7030 × 10^−1^**
$f_{9}$	Best	2.4809 × 10^3^	2.4810 × 10^3^	**2.4808 × 10^3^**	2.4810 × 10^3^	**2.4808 × 10^3^**	**2.4808 × 10^3^**	**2.4808 × 10^3^**	**2.4808 × 10^3^**
	Mean	2.5060 × 10^3^	2.5207 × 10^3^	2.4854 × 10^3^	2.5026 × 10^3^	**2.4808 × 10^3^**	**2.4808 × 10^3^**	**2.4808 × 10^3^**	**2.4808 × 10^3^**
	SD	3.0115 × 10^1^	3.6120 × 10^1^	2.5104 × 10^1^	1.9299 × 10^1^	6.0000 × 10^−4^	1.5000 × 10^−3^	9.0000 × 10^−4^	**0.0000**
$f_{10}$	Best	2.5005 × 10^3^	**2.5004 × 10^3^**	2.5005 × 10^3^	2.5013 × 10^3^	2.5005 × 10^3^	2.5005 × 10^3^	2.5505 × 10^3^	**2.5004 × 10^3^**
	Mean	3.3646 × 10^3^	3.4977 × 10^3^	3.7334 × 10^3^	4.4338 × 10^3^	3.8821 × 10^3^	2.5607 × 10^3^	**2.5351 × 10^3^**	2.5590 × 10^3^
	SD	9.0950 × 10^2^	6.9145 × 10^2^	9.9069 × 10^2^	1.0151 × 10^3^	8.2869 × 10^2^	1.7148 × 10^2^	**1.3649 × 10^3^**	1.4533 × 10^2^
$f_{11}$	Best	4.3921 × 10^3^	3.1346 × 10^3^	2.9000 × 10^3^	3.0195 × 10^3^	**2.60000 × 10^3^**	2.9024 × 10^3^	2.6019 × 10^3^	2.9000 × 10^3^
	Mean	5.3110 × 10^3^	3.6486 × 10^3^	2.9484 × 10^3^	4.2093 × 10^3^	2.9421 × 10^3^	2.9684 × 10^3^	2.9330 × 10^3^	**2.9250 × 10^3^**
	SD	5.2389 × 10^2^	3.8372 × 10^2^	8.8693 × 10^1^	7.1524 × 10^2^	1.1372 × 10^2^	1.3607 × 10^2^	1.0646 × 10^2^	**4.6803 × 10^1^**
$f_{12}$	Best	2.9487 × 10^3^	2.9486 × 10^3^	2.9430 × 10^3^	2.9618 × 10^3^	2.9379 × 10^3^	2.9366 × 10^3^	**2.9340 × 10^3^**	2.9348 × 10^3^
	Mean	2.9939 × 10^3^	2.9727 × 10^3^	2.9669 × 10^3^	3.0634 × 10^3^	2.9759 × 10^3^	2.9466 × 10^3^	**2.9412 × 10^3^**	2.9434 × 10^3^
	SD	2.9576 × 10^1^	1.7494 × 10^1^	2.7162 × 10^1^	7.7866 × 10^1^	4.7532 × 10^1^	1.2556 × 10^1^	5.1221	**5.1039**

**Table 4:** Wilcoxon rank-sum test results.

Functions	PSO	GWO	DBO	BKA	EDBO	SFOA	SFOAL
$f_{1}$	3.02 × 10^−11^	3.02 × 10^−11^	3.02 × 10^−11^	3.02 × 10^−11^	3.02 × 10^−11^	5.07 × 10^−10^	1.60 × 10^−3^
$f_{2}$	1.07 × 10^−7^	3.82 × 10^−11^	4.43 × 10^−3^	3.35 × 10^−8^	**5.01 × 10^−1^**	**1.96 × 10^−1^**	**5.19 × 10^−2^**
$f_{3}$	3.02 × 10^−11^	3.02 × 10^−11^	1.56 × 10^−8^	3.02 × 10^−11^	3.38 × 10^−8^	3.02 × 10^−11^	8.99 × 10^−11^
$f_{4}$	6.07 × 10^−11^	**2.90 × 10^−1^**	3.02 × 10^−11^	3.01 × 10^−7^	1.77 × 10^−3^	1.38 × 10^−2^	1.99 × 10^−2^
$f_{5}$	3.02 × 10^−11^	3.34 × 10^−11^	3.69 × 10^−11^	3.02 × 10^−11^	3.02 × 10^−11^	3.34 × 10^−11^	4.08 × 10^−11^
$f_{6}$	3.02 × 10^−11^	3.02 × 10^−11^	3.02 × 10^−11^	3.02 × 10^−11^	3.02 × 10^−11^	9.92 × 10^−11^	3.16 × 10^−10^
$f_{7}$	1.29 × 10^−9^	9.83 × 10^−8^	1.11 × 10^−6^	3.02 × 10^−11^	3.09 × 10^−6^	5.53 × 10^−8^	3.83 × 10^−5^
$f_{8}$	3.02 × 10^−11^	3.35 × 10^−8^	2.57 × 10^−7^	6.07 × 10^−11^	4.74 × 10^−6^	3.34 × 10^−11^	4.98 × 10^−11^
$f_{9}$	3.02 × 10^−11^	3.02 × 10^−11^	8.68 × 10^−3^	3.02 × 10^−11^	**2.06 × 10^−1^**	3.02 × 10^−11^	3.02 × 10^−11^
$f_{10}$	3.01 × 10^−7^	1.73 × 10^−6^	5.19 × 10^−7^	7.38 × 10^−10^	1.43 × 10^−8^	3.50 × 10^−3^	3.50 × 10^−3^
$f_{11}$	3.02 × 10^−11^	3.02 × 10^−11^	9.07 × 10^−3^	3.34 × 10^−11^	7.96 × 10^−3^	3.37 × 10^−5^	1.11 × 10^−4^
$f_{12}$	5.49 × 10^−11^	1.33 × 10^−10^	2.60 × 10^−8^	3.02 × 10^−11^	2.19 × 10^−8^	**9.00 × 10^−1^**	3.51 × 10^−2^

**Table 5:** Experimental results of incremental strategy variants.

Functions	Metrics	SR-SFOA	HADE-SFOA	Q-SFOA	T-SFOA	SFOA	QMSFOA
$f_{1}$	Best	3.1248 × 10^2^	**3.0013 × 10^2^**	3.1960 × 10^2^	3.4451 × 10^2^	3.4273 × 10^2^	3.0039 × 10^2^
	Mean	5.3385 × 10^2^	**3.0879 × 10^2^**	4.8474 × 10^2^	5.4644 × 10^2^	5.6335 × 10^2^	3.2321 × 10^2^
	SD	1.9276 × 10^2^	**1.2880 × 10^1^**	1.3782 × 10^2^	2.9671 × 10^2^	2.2323 × 10^2^	2.8478 × 10^1^
$f_{2}$	Best	4.0787 × 10^2^	4.4490 × 10^2^	4.0821 × 10^2^	4.0730 × 10^2^	4.0682 × 10^2^	**4.0424 × 10^2^**
	Mean	4.4729 × 10^2^	4.4903 × 10^2^	4.4677 × 10^2^	**4.4497 × 10^2^**	4.4548 × 10^2^	4.4796 × 10^2^
	SD	1.1038 × 10^1^	**4.7679**	7.4956	1.0398 × 10^1^	1.0510 × 10^1^	1.6619 × 10^1^
$f_{3}$	Best	6.0025 × 10^2^	**6.0003 × 10^2^**	6.0021 × 10^2^	6.0017 × 10^2^	6.0026 × 10^2^	**6.0003 × 10^2^**
	Mean	6.0061 × 10^2^	**6.0008 × 10^2^**	6.0058 × 10^2^	6.0061 × 10^2^	6.0066 × 10^2^	6.0010 × 10^2^
	SD	3.6830 × 10^−1^	**3.5900 × 10^−2^**	2.2400 × 10^−1^	3.2810 × 10^−1^	2.2680 × 10^−1^	5.2000 × 10^−2^
$f_{4}$	Best	8.3898 × 10^2^	**8.1492 × 10^2^**	8.2162 × 10^2^	8.2746 × 10^2^	8.2590 × 10^2^	8.1711 × 10^2^
	Mean	8.6581 × 10^2^	**8.3918 × 10^2^**	8.5416 × 10^2^	8.5721 × 10^2^	8.6646 × 10^2^	8.4956 × 10^2^
	SD	1.4391 × 10^1^	1.7045 × 10^1^	2.1426 × 10^1^	2.0071 × 10^1^	2.5164 × 10^1^	**1.3741 × 10^1^**
$f_{5}$	Best	9.0418 × 10^2^	**9.0000 × 10^2^**	9.0987 × 10^2^	9.0949 × 10^2^	9.0636 × 10^2^	**9.0000 × 10^2^**
	Mean	9.5735 × 10^2^	**9.0020 × 10^2^**	9.5214 × 10^2^	9.5145 × 10^2^	9.5761 × 10^2^	9.0041 × 10^2^
	SD	7.0394 × 10^1^	**5.4530 × 10^−1^**	5.2077 × 10^1^	3.5596 × 10^1^	4.7663 × 10^1^	1.3514
$f_{6}$	Best	1.8590 × 10^3^	1.8566 × 10^3^	1.8784 × 10^3^	1.8551 × 10^3^	1.8764 × 10^3^	**1.8131 × 10^3^**
	Mean	1.9435 × 10^3^	2.0154 × 10^3^	2.0536 × 10^3^	1.9299 × 10^3^	1.9687 × 10^3^	**1.8475 × 10^3^**
	SD	8.9366 × 10^1^	5.3395 × 10^2^	5.6020 × 10^2^	5.5866 × 10^1^	7.8783 × 10^1^	**2.2631 × 10^1^**
$f_{7}$	Best	2.0412 × 10^3^	2.0291 × 10^3^	2.0478 × 10^3^	2.0344 × 10^3^	2.0372 × 10^3^	**2.0284 × 10^3^**
	Mean	2.0607 × 10^3^	2.0462 × 10^3^	2.0663 × 10^3^	2.0579 × 10^3^	2.0591 × 10^3^	**2.0407 × 10^3^**
	SD	9.6791	2.1583 × 10^1^	1.2184 × 10^1^	1.5467 × 10^1^	1.2470 × 10^1^	**7.2294**
$f_{8}$	Best	2.2268 × 10^3^	2.2274 × 10^3^	2.2284 × 10^3^	2.2265 × 10^3^	2.2265 × 10^3^	**2.2220 × 10^3^**
	Mean	2.2309 × 10^3^	2.2296 × 10^3^	2.2328 × 10^3^	2.2308 × 10^3^	2.2293 × 10^3^	**2.2247 × 10^3^**
	SD	2.1041	1.5363	3.9010	2.6228	1.2586	**9.7030 × 10^−1^**
$f_{9}$	Best	**2.4808 × 10^3^**	**2.4808 × 10^3^**	**2.4808 × 10^3^**	**2.4808 × 10^3^**	**2.4808 × 10^3^**	**2.4808 × 10^3^**
	Mean	**2.4808 × 10^3^**	**2.4808 × 10^3^**	**2.4808 × 10^3^**	**2.4808 × 10^3^**	**2.4808 × 10^3^**	**2.4808 × 10^3^**
	SD	2.0000 × 10^−4^	**0.0000**	2.0000 × 10^−4^	2.0000 × 10^−4^	1.5000 × 10^−3^	**0.0000**
$f_{10}$	Best	**2.5004 × 10^3^**	2.5005 × 10^3^	2.5006 × 10^3^	**2.5004 × 10^3^**	2.5005 × 10^3^	**2.5004 × 10^3^**
	Mean	2.6627 × 10^3^	2.9212 × 10^3^	2.8603 × 10^3^	2.9240 × 10^3^	2.5607 × 10^3^	**2.5590 × 10^3^**
	SD	3.6534 × 10^2^	5.1292 × 10^2^	4.6556 × 10^2^	4.9040 × 10^2^	1.7148 × 10^2^	**1.4533 × 10^2^**
$f_{11}$	Best	2.9007 × 10^3^	2.9000 × 10^3^	2.9032 × 10^3^	**2.6736 × 10^3^**	2.9024 × 10^3^	2.9000 × 10^3^
	Mean	2.9220 × 10^3^	**2.9000 × 10^3^**	2.9268 × 10^3^	2.9186 × 10^3^	2.9684 × 10^3^	2.9250 × 10^3^
	SD	5.7644 × 10^1^	**5.1000 × 10^−3^**	5.7353 × 10^1^	7.8516 × 10^1^	1.3607 × 10^2^	4.6803 × 10^1^
$f_{12}$	Best	2.9341 × 10^3^	**2.9303 × 10^3^**	2.9340 × 10^3^	2.9357 × 10^3^	2.9366 × 10^3^	2.9348 × 10^3^
	Mean	2.9411 × 10^3^	2.9429 × 10^3^	**2.9409 × 10^3^**	2.9422 × 10^3^	2.9466 × 10^3^	2.9434 × 10^3^
	SD	4.2461	7.0791	3.7189	**3.1174**	1.2560 × 10^1^	5.1039

**Table 6:** Experimental results of removal strategy variants.

Functions	Metrics	QMSFOA -NoSR	QMSFOA -NoHADE	QMSFOA -NoQL	QMSFOA -NoTD	SFOA	QMSFOA
$f_{1}$	Best	3.0127 × 10^2^	3.3129 × 10^2^	3.0107 × 10^2^	**3.0011 × 10^2^**	3.4273 × 10^2^	3.0039 × 10^2^
	Mean	3.3577 × 10^2^	5.0341 × 10^2^	3.4430 × 10^2^	3.2743 × 10^2^	5.6335 × 10^2^	**3.2321 × 10^2^**
	SD	4.7838 × 10^1^	1.5511 × 10^2^	7.8607 × 10^1^	2.9833 × 10^1^	2.2323 × 10^2^	**2.8478 × 10^1^**
$f_{2}$	Best	4.0464 × 10^2^	4.0566 × 10^2^	4.0020 × 10^2^	**4.0008 × 10^2^**	4.0682 × 10^2^	4.0424 × 10^2^
	Mean	4.4682 × 10^2^	4.4839 × 10^2^	**4.4082 × 10^2^**	4.4672 × 10^2^	4.4548 × 10^2^	4.4796 × 10^2^
	SD	1.2273 × 10^1^	1.0634 × 10^1^	1.7069 × 10^1^	1.7840 × 10^1^	**1.0510 × 10^1^**	1.6619 × 10^1^
$f_{3}$	Best	6.0004 × 10^2^	6.0023 × 10^2^	**6.0002 × 10^2^**	6.0003 × 10^2^	6.0026 × 10^2^	6.0003 × 10^2^
	Mean	6.0010 × 10^2^	6.0055 × 10^2^	6.0010 × 10^2^	**6.0009 × 10^2^**	6.0066 × 10^2^	6.0010 × 10^2^
	SD	6.6700 × 10^−2^	2.3300 × 10^−1^	4.9100 × 10^−2^	**3.4400 × 10 ^−^^2^**	2.2680 × 10^−1^	5.2000 × 10^−2^
$f_{4}$	Best	8.2189 × 10^2^	8.2396 × 10^2^	8.2531 × 10^2^	8.2313 × 10^2^	8.2590 × 10^2^	**8.1711 × 10^2^**
	Mean	**8.4122 × 10^2^**	8.6536 × 10^2^	8.4857 × 10^2^	8.4828 × 10^2^	8.6646 × 10^2^	8.4956 × 10^2^
	SD	**1.2002 × 10^1^**	1.7167 × 10^1^	1.6127 × 10^1^	1.6203 × 10^1^	2.5164 × 10^1^	1.3741 × 10^1^
$f_{5}$	Best	**9.0000 × 10^2^**	9.0290 × 10^2^	**9.0000 × 10^2^**	**9.0000 × 10^2^**	9.0636 × 10^2^	**9.0000 × 10^2^**
	Mean	9.0038 × 10^2^	9.2466 × 10^2^	9.0016 × 10^2^	**9.0013 × 10^2^**	9.5761 × 10^2^	9.0041 × 10^2^
	SD	1.6375	1.5622 × 10^1^	5.0850 × 10^−1^	**2.2400 × 10 ^−^^1^**	4.7663 × 10^1^	1.3514
$f_{6}$	Best	**1.8083 × 10^3^**	1.8390 × 10^3^	1.8143 × 10^3^	1.8111 × 10^3^	1.8764 × 10^3^	1.8131 × 10^3^
	Mean	1.9365 × 10^3^	1.8894 × 10^3^	1.8777 × 10^3^	1.9094 × 10^3^	1.9687 × 10^3^	**1.8475 × 10^3^**
	SD	4.3852 × 10^2^	3.4975 × 10^1^	1.0927 × 10^2^	2.2887 × 10^2^	7.8783 × 10^1^	**2.2631 × 10^1^**
$f_{7}$	Best	2.0310 × 10^3^	2.0276 × 10^3^	**2.0265 × 10^3^**	2.0272 × 10^3^	2.0372 × 10^3^	2.0284 × 10^3^
	Mean	2.0405 × 10^3^	2.0505 × 10^3^	**2.0396 × 10^3^**	2.0429 × 10^3^	2.0591 × 10^3^	2.0407 × 10^3^
	SD	9.4888	1.0518 × 10^1^	9.5320	9.2001	1.2470 × 10^1^	**7.2294**
$f_{8}$	Best	2.2227 × 10^3^	2.2230× 10^3^	2.2227 × 10^3^	2.2252 × 10^3^	2.2265 × 10^3^	**2.2220 × 10^3^**
	Mean	2.2254 × 10^3^	2.2257 × 10^3^	**2.2244 × 10^3^**	2.2262 × 10^3^	2.2293 × 10^3^	2.2247 × 10^3^
	SD	1.1091	1.1801	9.8710 × 10^−1^	**6.4300 × 10 ^−^^1^**	1.2586	9.7030 × 10^−1^
$f_{9}$	Best	**2.4808 × 10^3^**	**2.4808 × 10^3^**	**2.4808 × 10^3^**	**2.4808 × 10^3^**	**2.4808 × 10^3^**	**2.4808 × 10^3^**
	Mean	**2.4808 × 10^3^**	**2.4808 × 10^3^**	**2.4808 × 10^3^**	**2.4808 × 10^3^**	**2.4808 × 10^3^**	**2.4808 × 10^3^**
	SD	**0.0000**	6.0000 × 10^−4^	**0.0000**	1.0000 × 10^−4^	1.5000 × 10^−3^	**0.0000**
$f_{10}$	Best	**2.5004 × 10^3^**	**2.5004 × 10^3^**	2.5005 × 10^3^	2.5006 × 10^3^	2.5005 × 10^3^	**2.5004 × 10^3^**
	Mean	2.5586 × 10^3^	**2.5509 × 10^3^**	2.6230 × 10^3^	2.6382 × 10^3^	2.5607 × 10^3^	2.5590 × 10^3^
	SD	1.0676 × 10^2^	**9.8279 × 10^1^**	2.1344 × 10^2^	1.5911 × 10^2^	1.7148 × 10^2^	1.4533 × 10^2^
$f_{11}$	Best	**2.9000 × 10^3^**	2.9004 × 10^3^	**2.9000 × 10^3^**	2.9000 × 10^3^	2.9024 × 10^3^	**2.9000 × 10^3^**
	Mean	**2.9000 × 10^3^**	2.9463 × 10^3^	2.9167 × 10^3^	2.9068 × 10^3^	2.9684 × 10^3^	2.9250 × 10^3^
	SD	**1.0000 × 10 ^−^^2^**	9.6607 × 10^1^	3.7918 × 10^1^	2.5683 × 10^1^	1.3607 × 10^2^	4.6803 × 10^1^
$f_{12}$	Best	2.9346 × 10^3^	2.9338 × 10^3^	**2.9333 × 10^3^**	2.9340 × 10^3^	2.9366 × 10^3^	2.9348 × 10^3^
	Mean	2.9465 × 10^3^	**2.9427 × 10^3^**	2.9485 × 10^3^	2.9477 × 10^3^	2.9466 × 10^3^	2.9434 × 10^3^
	SD	9.9883	6.6452	1.1552 × 10^1^	1.4010 × 10^1^	1.2560 × 10^1^	**5.1039**

**Table 7:** Results across four aeromagnetic missions.

Missions	Metrics	PSO	GWO	DBO	BKA	EDBO	SFOA	SFOAL	QMSFOA
NO.1	Best	1.0574 × 10^3^	8.4660 × 10^2^	7.9212 × 10^2^	**7.7015 × 10^2^**	8.0537 × 10^2^	1.2159 × 10^3^	9.4601 × 10^2^	8.2488 × 10^2^
	Mean	1.8855 × 10^3^	2.1372 × 10^3^	2.8638 × 10^3^	2.1679 × 10^3^	1.5523 × 10^3^	1.4335 × 10^3^	1.3893 × 10^3^	**9.0133 × 10^2^**
	SD	7.5383 × 10^2^	1.6439 × 10^3^	2.0603 × 10^3^	1.0043 × 10^3^	4.4085 × 10^2^	1.6979 × 10^2^	2.3555 × 10^2^	**7.5366 × 10^1^**
NO.2	Best	7.0724 × 10^3^	6.3584 × 10^3^	6.2893 × 10^3^	5.8305 × 10^3^	5.8111 × 10^3^	5.9722 × 10^3^	6.8693 × 10^3^	**5.5539 × 10^3^**
	Mean	9.4869 × 10^3^	9.2552 × 10^3^	1.1085 × 10^4^	8.1368 × 10^3^	8.3518 × 10^3^	7.4209 × 10^3^	7.1902 × 10^3^	**6.2932 × 10^3^**
	SD	1.4824 × 10^3^	2.8665 × 10^3^	3.3642 × 10^3^	1.9343 × 10^3^	1.9368 × 10^3^	6.6697 × 10^2^	6.1364 × 10^2^	**2.7343 × 10^3^**
NO.3	Best	1.4722 × 10^4^	1.7257 × 10^4^	1.2350 × 10^4^	1.5111 × 10^4^	1.3273 × 10^4^	1.3944 × 10^4^	1.4836 × 10^4^	**1.2265 × 10^4^**
	Mean	1.6911 × 10^4^	1.9598 × 10^4^	1.6392 × 10^4^	2.2347 × 10^4^	2.2556 × 10^4^	1.5276 × 10^4^	1.6614 × 10^4^	**1.3473 × 10^4^**
	SD	1.3421 × 10^3^	2.2597 × 10^3^	3.3396 × 10^3^	6.8539 × 10^3^	8.3161 × 10^3^	9.1775 × 10^2^	**8.7453 × 10^2^**	1.1200 × 10^3^
NO.4	Best	4.5181 × 10^3^	5.5329 × 10^3^	4.9236 × 10^3^	4.5514 × 10^3^	**3.2308 × 10^3^**	5.5513 × 10^3^	5.0380 × 10^3^	4.0584 × 10^3^
	Mean	8.2587 × 10^3^	8.2677 × 10^3^	1.0075 × 10^4^	1.2662 × 10^4^	6.6680 × 10^3^	6.8091 × 10^3^	7.6809 × 10^3^	**4.7390 × 10^3^**
	SD	2.9821 × 10^3^	4.7244 × 10^3^	4.3227 × 10^3^	5.2704 × 10^3^	2.2481 × 10^3^	8.5395 × 10^2^	1.3291 × 10^3^	**5.4756 × 10^2^**

## Data Availability

All data in this paper are included in the manuscript.

## Abbreviations

Notation table.

**Notation**	**Description**
N	Population size
D	Dimensionality of the optimization problem
$T,T_{max}$	Current iteration number and the maximum number of iterations
$X_{i,j}$	Position of the i-th individual in the j-th dimension
$X_{best}$	Position of the current global best solution
$l_{b},\;$$u_{b}$	Lower and upper bounds of the search variables
$E_{t}$	Starfish’s energy during the exploration phase
$s_{i,j}$	Quasi-random number generated by the Sobol sequence
η, γ	Scaling factor and refractive index in ROBL
$CR_{i,t}$	Adaptive dimension selection rate for the i-th individual
$\mu_{CR},\;\sigma_{CR}$	Mean and standard deviation of CR
$\mu_{F},\;\sigma_{F}$	Location and scale parameters of the Cauchy distribution
$P_{i,t}$	Selection probability for mutation strategies
$R_{k}$	Success rate of the k-th mutation strategy
$\xi_{t}$	Iteration progress in Q-learning state space
$Div_{t}$	Population diversity metric in Q-learning state space
$Q\left(s_{t},a_{t}\right)$	Action-value function for state $s_{t}$ and action $a_{t}$
$r_{t}$	Reward function at iteration t
α,γ	Learning rate and discount factor in Q-learning
ε	Exploration rate in the ε-greedy strategy
$c_{stag}$	Consecutive stagnation count
$\nu_{t}$	Dynamic degree of freedom in the adaptive t-distribution
$\lambda_{t}$	Dynamic step size factor
$Z_{b}\left(x,y\right)$	Base terrain generated by fractal noise
$Z_{f}\left(x,y\right)$	Final 3D simulation terrain model height
ρ, τ	Persistence and lacunarity controlling terrain texture
$H_{w}$	Water level threshold for clamping terrain
$\Omega_{tree},\Omega_{tower}$	Spatial sets of vegetation and transmission towers
$L_{obs},L_{uav}$	Line segments representing obstacle cables and UAV flight
$Z_{i},Z_{f,i}$	Absolute flight altitude and corresponding terrain elevation
$h_{i},h_{o}$	Actual ground clearance and optimal detection clearance
$\left[h_{min},h_{max}\right]$	Bounds of the safe flight corridor
$D_{1},D_{2}$	Safety buffer distances for cylindrical and linear obstacles
$\varphi_{i},\Psi_{i}$	Horizontal turning angle and vertical pitch angle
$L_{ref},d_{i}$	Preset survey line and perpendicular cross-track error

## References

[1] Accomando F., Barone A., Mercogliano F., Milano M., Vitale A., Castaldo R., Tizzani P. (2025). Advances in Magnetic UAV Sensing: A Comparative Study of the MagNimbus and MagArrow Magnetometers. Sensors.

[2] Walter C., Braun A., Fotopoulos G. (2020). High-Resolution Unmanned Aerial Vehicle Aeromagnetic Surveys for Mineral Exploration Targets. Geophys. Prospect..

[3] Takáč M., Kletetschka G., Petrucha V. (2025). UAV aeromagnetic survey of the Acraman impact structure: Insights into the central magnetic anomaly. Front. Earth Sci..

[4] Aali A.A., Shirazy A., Shirazi A., Pour A.B., Hezarkhani A., Maghsoudi A., Hashim M., Khakmardan S. (2022). Fusion of Remote Sensing, Magnetometric, and Geological Data to Identify Polymetallic Mineral Potential Zones in Chakchak Region, Yazd, Iran. Remote Sens..

[5] Chavanidis K., Salem A., Stampolidis A., Ashadi A.L., Abu-Mahfouz I.S., Kirmizakis P., Soupios P. (2024). Aeromagnetic Data Analysis of Geothermal Energy Potential of a Hot Spring Area in Western Saudi Arabia. Nat. Resour. Res..

[6] Nikulin A., de Smet T.S. (2023). UAV-Based Aeromagnetic Surveys for Orphaned Well Location: Emerging Best Practices. Lead. Edge.

[7] Wu X., Xue G.-Q., Wang Y.-B., Cui S. (2025). Current Progress in and Future Visions of Key Technologies of UAV-Borne Multi-Modal Geophysical Exploration for Mineral Exploration: A Scoping Review. Remote Sens..

[8] Betts P.G., Moore D., Aitken A., Blaikie T., Jessell M., Ailleres L., Armit R., McLean M., Munukutla R., Chukwu C. (2024). Geology from Aeromagnetic Data. Earth-Sci. Rev..

[9] Parshin A.V., Morozov V.A., Blinov A.V., Kosterev A.N., Budyak A.E. (2018). Low-Altitude Geophysical Magnetic Prospecting Based on Multirotor UAV as a Promising Replacement for Traditional Ground Survey. Geo-Spat. Inf. Sci..

[10] Zheng Y., Li S., Xing K., Zhang X. (2021). Unmanned Aerial Vehicles for Magnetic Surveys: A Review on Platform Selection and Interference Suppression. Drones.

[11] Li Z., Gao S. (2018). New method of aeromagnetic surveys with rotorcraft UAV in particular areas. Chin. J. Geophys..

[12] Li H., Luo J., Zhang J., Li J., Zhang Y., Zhang W., Zhang M. (2024). Determinants of Maximum Magnetic Anomaly Detection Distance. Sensors.

[13] Perikleous D., Margariti K., Velanas P., Blazquez C.S., Garcia P.C., Gonzalez-Aguilera D. (2024). Application of Magnetometer-Equipped Drone for Mineral Exploration in Mining Operations. Drones.

[14] Zhang N., Xu L.-Z., An T.-H., Zhang J.-G., Guo H. (2025). Unmanned Aerial Vehicle (UAV) Aeromagnetic System Based on Fluxgate Sensor and Its Applications. Appl. Geophys..

[15] Feng Y., Yang J., Wang Y., Cong K., Wang L., Zu Z. Efficient Terrain-Following Path Planning and Trajectory Tracking Control for Fixed-Wing UAV. Proceedings of the 2025 IEEE 20th Conference on Industrial Electronics and Applications (ICIEA).

[16] Schmidt V., Becken M., Schmalzl J. (2020). A UAV-Borne Magnetic Survey for Archaeological Prospection of a Celtic Burial Site. First Break.

[17] Kaub L., Keller G., Bouligand C., Glen J.M.G. (2021). Magnetic Surveys with Unmanned Aerial Systems: Software for Assessing and Comparing the Accuracy of Different Sensor Systems, Suspension Designs and Compensation Methods. Geochem. Geophys. Geosystems.

[18] Yuan G. (2025). An Aeromagnetic Compensation Algorithm Based on Complete Ensemble Empirical Mode Decomposition with Adaptive Noise and a Physics-Guided Neural Network. Fuel Cells Bull..

[19] Liu L., Ru L., Wang W., Xi H., Zhu R., Li S., Zhang Z. (2025). UAV Path Planning in Threat Environment: A*-APF Algorithm for Spatio-Temporal Grid Optimization. Drones.

[20] Hao G., Lv Q., Huang Z., Zhao H., Chen W. (2023). UAV Path Planning Based on Improved Artificial Potential Field Method. Aerospace.

[21] Huang T., Fan K., Sun W., Li W., Guo H. (2023). Potential-Field-RRT: A Path-Planning Algorithm for UAVs Based on Potential-Field-Oriented Greedy Strategy to Extend Random Tree. Drones.

[22] Ezugwu A.E., Shukla A.K., Nath R., Akinyelu A.A., Agushaka J.O., Chiroma H., Muhuri P.K. (2021). Metaheuristics: A Comprehensive Overview and Classification along with Bibliometric Analysis. Artif. Intell. Rev..

[23] Wu Q., Su Y., Tan W., Zhan R., Liu J., Jiang L. (2025). UAV Path Planning Trends from 2000 to 2024: A Bibliometric Analysis and Visualization. Drones.

[24] Hu W., Ma X. (2025). Optimization Algorithm of UAVs Task Assignment and Path Planning Based on Dynamic Cluster Particle Swarm Optimization. IEEE Trans. Intell. Transp. Syst..

[25] Mai X., Dong N., Liu S., Chen H. (2023). UAV path planning based on a dual-strategy ant colony optimization algorithm. OAE Publ. Inc..

[26] Chai X., Zhou G., Wang H., Liu P., Liu Z., Li C., Yan L. (2025). UAV Path Planners in Complex Environments: A Multi-Dimensional Perturbation Based on Artificial Bee Colony. IEEE Access.

[27] Qu C., Gai W., Zhang J., Zhong M. (2020). A Novel Hybrid Grey Wolf Optimizer Algorithm for Unmanned Aerial Vehicle (UAV) Path Planning. Knowl.-Based Syst..

[28] Yang Y., Fu Y., Lu D., Xiang H., Xu K. (2024). Three-Dimensional Unmanned Aerial Vehicle Trajectory Planning Based on the Improved Whale Optimization Algorithm. Symmetry.

[29] Shehab M., Mashal I., Momani Z., Shambour M.K.Y., Al-Badareen A., Al-Dabet S., Bataina N., Alsoud A.R., Abualigah L. (2022). Harris Hawks Optimization Algorithm: Variants and Applications. Arch. Comput. Methods Eng..

[30] Xue J., Shen B. (2020). A Novel Swarm Intelligence Optimization Approach: Sparrow Search Algorithm. Syst. Sci. Control. Eng..

[31] Xue J., Shen B. (2022). Dung Beetle Optimizer: A New Meta-Heuristic Algorithm for Global Optimization. J. Supercomput..

[32] Trojovský P., Dehghani M. (2022). Pelican Optimization Algorithm: A Novel Nature-Inspired Algorithm for Engineering Applications. Sensors.

[33] Wang J., Wang W.-C., Hu X.-X., Qiu L., Zang H.-F. (2024). Black-Winged Kite Algorithm: A Nature-Inspired Meta-Heuristic for Solving Benchmark Functions and Engineering Problems. Artif. Intell. Rev..

[34] Hussain K., Mohd Salleh M.N., Cheng S., Shi Y. (2018). Metaheuristic Research: A Comprehensive Survey. Artif. Intell. Rev..

[35] Yang Y., He Q., Yang L. (2022). UAV Trajectory Planning Based on an Improved Sparrow Optimization Algorithm with Multi-Strategy Integration. Front. Environ. Sci..

[36] Qiu S., Dai J., Zhao D. (2024). Path Planning of an Unmanned Aerial Vehicle Based on a Multi-Strategy Improved Pelican Optimization Algorithm. Biomimetics.

[37] Tu K., Cheng J. (2025). Enhanced Dung Beetle Optimization Algorithm and Its Application in 3D UAV Path Planning. Electron. Res. Arch..

[38] Zhong C., Li G., Meng Z., Li H., Yildiz A.R., Mirjalili S. (2024). Starfish Optimization Algorithm (SFOA): A Bio-Inspired Metaheuristic Algorithm for Global Optimization Compared with 100 Optimizers. Neural Comput. Appl..

[39] Zheng W., Ai Y., Zhang W. (2024). Improved Snake Optimizer Using Sobol Sequential Nonlinear Factors and Different Learning Strategies and Its Applications. Mathematics.

[40] Xiao Y., Sun X., Guo Y., Cui H., Wang Y., Li J., Li S. (2022). An Enhanced Honey Badger Algorithm Based on Lévy Flight and Refraction Opposition-Based Learning for Engineering Design Problems. J. Intell. Fuzzy Syst..

[41] Tubishat M., Idris N., Shuib L., Abushariah M.A., Mirjalili S. (2020). Improved Salp Swarm Algorithm Based on Opposition Based Learning and Novel Local Search Algorithm for Feature Selection. Expert Syst. Appl..

[42] Ahmad M.F., Isa N.A.M., Lim W.H., Ang K.M. (2022). Differential Evolution: A Recent Review Based on State-of-the-Art Works. Alex. Eng. J..

[43] Qin A.K., Suganthan P.N. Self-Adaptive Differential Evolution Algorithm for Numerical Optimization. Proceedings of the 2005 IEEE Congress on Evolutionary Computation.

[44] Zhang J., Sanderson A.C. (2009). JADE: Adaptive Differential Evolution with Optional External Archive. IEEE Trans. Evol. Comput..

[45] Peng Q., Gao K., Fu Y., Li J., Rahman H.F. (2025). Integrating Meta-Heuristics and Q-Learning for Solving Hybrid Flow Shop Scheduling and Rescheduling Problems with Reentrant. J. Ind. Manag. Optim..

[46] Accomando F., Florio G. (2024). Applicability of Small and Low-Cost Magnetic Sensors to Geophysical Exploration. Sensors.

[47] Palumbo D., Bignardi S., Menozzi O., Staffilani P., Pepe M. (2026). When Geophysics Meets Geomatics and Archeology: Revealing the Connection Between Surface and Buried Structures at Iuvanum Archeological Site. Remote Sens..

[48] Husain A., Nanda M.N., Chowdary M.S., Sajid M. (2022). Fractals: An Eclectic Survey, Part II. Fractal Fract..

[49] Zou Z., Du Y., Song H. (2023). Fractal Features in Terrain Restoration of Jiuzhai Valley, a World Natural Heritage Site in China. Fractal Fract..

[50] Zheng X., Bao Z., Yin Q. (2023). Terrain Self-Similarity-Based Transformer for Generating Super Resolution DEMs. Remote Sens..

[51] Xiao E., Qin L., Chi Z., Gu H., Hua Y., Yang H., Li R. (2026). Research on Seafloor 3D Reconstruction Method Based on Sparse Measurement Points. Sensors.

[52] Pastucha E., Puniach E., Ścisłowicz A., Ćwiąkała P., Niewiem W., Wiącek P. (2020). 3D Reconstruction of Power Lines Using UAV Images to Monitor Corridor Clearance. Remote Sens..

[53] Jakob W., Blume C. (2014). Pareto Optimization or Cascaded Weighted Sum: A Comparison of Concepts. Algorithms.

[54] Quadt T., Lindelauf R., Voskuijl M., Monsuur H., Čule B. (2024). Dealing with Multiple Optimization Objectives for UAV Path Planning in Hostile Environments: A Literature Review. Drones.

[55] Zhai L., Wu H., Lai L., Gao Z. (2025). Intelligent Optimization Algorithms for Multi-UAV Path Planning: A Comprehensive Review. IEEE Access.

[56] Li C., Zhao Q., Che C. (2025). 3D Flight Path Planning for UAV Based on Improved Particle Swarm Optimization Algorithm. IEEE Access.

[57] Liu Y., Fu M., Liu Z., Liu H., Peng W., Li L., Yang Y., Zhou X., Jia C. (2025). An Enhanced Starfish Optimization Algorithm via Joint Strategy and Its Application in Ultra-Wideband Indoor Positioning. Biomimetics.

[58] Song C., Zhang X., She Y., Li B., Zhang Q. (2025). Trajectory Planning for UAV Swarm Tracking Moving Target Based on an Improved Model Predictive Control Fusion Algorithm. IEEE Internet Things J..

[59] Luo X., Wang Q., Gong H., Tang C. (2024). UAV Path Planning Based on the Average TD3 Algorithm with Prioritized Experience Replay. IEEE Access.

[60] Choi J., Lee D., Lee I. (2025). Model Predictive Control of Aerial Manipulation via Hardware-in-the-Loop Simulation. IEEE Access.

